# A global dataset of the fraction of absorbed photosynthetically active radiation for 1982–2022

**DOI:** 10.1038/s41597-024-03561-0

**Published:** 2024-06-28

**Authors:** Weiqing Zhao, Zaichun Zhu, Sen Cao, Muyi Li, Junjun Zha, Jiabin Pu, Ranga B. Myneni

**Affiliations:** 1 School of Urban Planning and Design, Shenzhen Graduate School, Peking University, Shenzhen, 518055 China; 2 Institute of Carbon Neutrality, Peking University, Beijing, 100871 China; 3 Key Laboratory of Earth Surface System and Human—Earth Relations, Ministry of Natural Resources of China, Shenzhen Graduate School, Peking University, Shenzhen, 518055 China; 4 Department of Earth and Environment, Boston University, Boston, MA 02215 USA

**Keywords:** Carbon cycle, Ecology

## Abstract

The fraction of absorbed photosynthetically active radiation (FPAR) is an essential biophysical parameter that characterizes the structure and function of terrestrial ecosystems. Despite the extensive utilization of several satellite-derived FPAR products, notable temporal inconsistencies within each product have been underscored. Here, the new generation of the GIMMS FPAR product, GIMMS FPAR4g, was developed using a combination of a machine learning algorithm and a pixel-wise multi-sensor records integration approach. PKU GIMMS NDVI, which eliminates the orbital drift and sensor degradation issues, was used as the data source. Comparisons with ground-based measurements indicate root mean square errors ranging from 0.10 to 0.14 with R-squared ranging from 0.73 to 0.87. More importantly, our product demonstrates remarkable spatiotemporal coherence and continuity, revealing a persistent terrestrial darkening over the past four decades (0.0004 yr^−1^, p < 0.001). The GIMMS FPAR4g, available for half-month intervals at a spatial resolution of 1/12° from 1982 to 2022, promises to be a valuable asset for in-depth analyses of vegetation structures and functions spanning the last 40 years.

## Background & Summary

Photosynthesis is a critical physiological process for vegetation and strongly mediates the global carbon cycle^1,2^. The rate of photosynthesis in plants depends on the energy absorbed by the vegetation^3^. Hence, the fraction of absorbed photosynthetically active radiation (FPAR) has been introduced to quantitatively characterize the capacity of vegetation canopy to absorb PAR^3,4^. It is defined as the fraction of PAR absorbed by vegetation within the 400–700 nm wavelength range^5^. FPAR, a biophysical variable with a clear meaning, has been designated as one of the essential climate variables by the Global Climate Observing System (GCOS)^6^. As a variable that bridges ecosystem function and structure, it has been applied in various light use efficiency (LUE) models to estimate vegetation productivity since the 1990s^7–10^. Furthermore, FPAR serves as a key state variable in many climate, hydrological, and ecological models for describing biogeophysical and biogeochemical processes^11–13^. It has been extensively employed in research to investigate the interactions between vegetation, climate system, and human society^14–16^.

A continuous record of FPAR is crucial for understanding how terrestrial ecosystems respond to and provide feedback on global environmental changes^17–19^. New and advanced sensors such as the Moderate Resolution Imaging Spectroradiometer (MODIS), the Visible Infrared Imager Radiometer Suite (VIIRS), and Système Pour l’Observation de la Terre VEGETATION (SPOT-VGT) provide us with several sets of global FPAR products with high spatiotemporal resolution. However, substantial disparities among FPAR products stem from variations in retrieval algorithms and sensors^20^, the magnitude of which manifests differently across distinct land cover types. An analysis focusing on Northern Eurasia in 2000 spotlighted pronounced disparities in grasslands, shrublands, and needleleaf forests among four FPAR datasets (MODIS, SeaWiFS, Carbon cycle and Change in Land Observational Products from an Ensemble of Satellites [CYCLOPES], and GLOBCARBON)^21^. Nevertheless, the limited temporal span of these products highlights the importance of long-term global FPAR records. The Advanced Very High-Resolution Radiometer (AVHRR) sensors onboard the National Oceanic and Atmospheric Administration (NOAA) satellite series since the 1980s have been the only source of such global FPAR time series. However, these AVHRR-based datasets are also not free from problems related to retrieval algorithms and calibration^22^. Xiao *et al*.^23^ underscored that AVHRR-based FPAR products (Global Inventory Monitoring and Modeling System [GIMMS] FPAR3g, Global Land Surface Satellite [GLASS] AVHRR FPAR, and National Centers for Environmental Information [NCEI] AVHRR FPAR) reveal inconsistencies, particularly in tropical forests and sparsely vegetated zones. More importantly, the AVHRR series are tainted with challenges like orbit drifts, sensor degradation, and platform replacements, inducing potential temporal inconsistencies within the products themselves^24,25^. The GCOS has specified criteria concerning the measurement uncertainty and stability of FPAR products. Nevertheless, the scarcity of ground-based FPAR measurements impedes the validation of FPAR products predating 2000. Against this backdrop, the issue of accuracy assessment and validation of temporal consistency for existing FPAR datasets urgently needs to be addressed.

The uncertainty in satellite-derived FPAR datasets directly affects research related to estimating ecosystem productivity, global carbon cycle simulations, and insights into ecosystem structure and function^26–29^. The uncertainty in FPAR as a critical input parameter may introduce notable errors in carbon and water model simulations^30–33^. Substantial discrepancies among FPAR products in magnitude, as well as the start and end of the growing season, directly affect the evaluation and improvement of dynamic global vegetation models, which could result in diverse effects on the simulation of the carbon cycle^34–36^. Furthermore, FPAR is also employed to assess the variability in ecosystem structure and function. Research at mixed forest sites revealed a sharp decrease in the variability of GIMMS FPAR3g with increasing timescale, indicating a rapid ecosystem recovery rate after disturbances^37^. However, it is not observed in MODIS Two–stream Inversion Package FPAR. The uncertainty in FPAR data hinders our understanding of ecosystem stability and resilience. More critically, a range of well-documented issues related to platforms/sensors has resulted in pervasive temporal inconsistencies in products developed from AVHRR observation data^24,38^. This has not only sparked extensive debates regarding global vegetation’s greening^39,40^ but also introduced substantial uncertainty into the studies of other long-term trends^41–44^. To accurately understand global vegetation changes, there is a pressing need for a satellite-derived capable of accurately mapping global FPAR over the past 40 years, offering consistent spatiotemporal observations that can be validated across their entire duration.

This research introduces the new generation of the GIMMS FPAR dataset - the GIMMS FPAR4g. Firstly, we trained Back Propagation Neural Network (BPNN) models for each half-month using PKU GIMMS NDVI^45^ and Sensor-Independent (SI) FPAR Climate Data Record (CDR) and developed the FPAR data spanning from 1982 to 2015, herein referred to as FPAR4g solely. Subsequently, we extended the temporal span of the GIMMS FPAR4g (1982–2022) by integrating the SI FPAR and the FPAR4g solely using the pixel-wise regression approach. Finally, we evaluated the accuracy of our product through direct comparisons with extensive Landsat FPAR reference samples and ground-based measurements, as well as indirect comparisons with other FPAR satellite-derived products. The final GIMMS FPAR4g product features a 15-day temporal resolution and a spatial resolution of 1/12 degree, covering four decades from 1982 to 2022, and importantly, it exhibits strong temporal consistency across the pre- and post-2000 periods.

## Methods

**Fig. 1:**
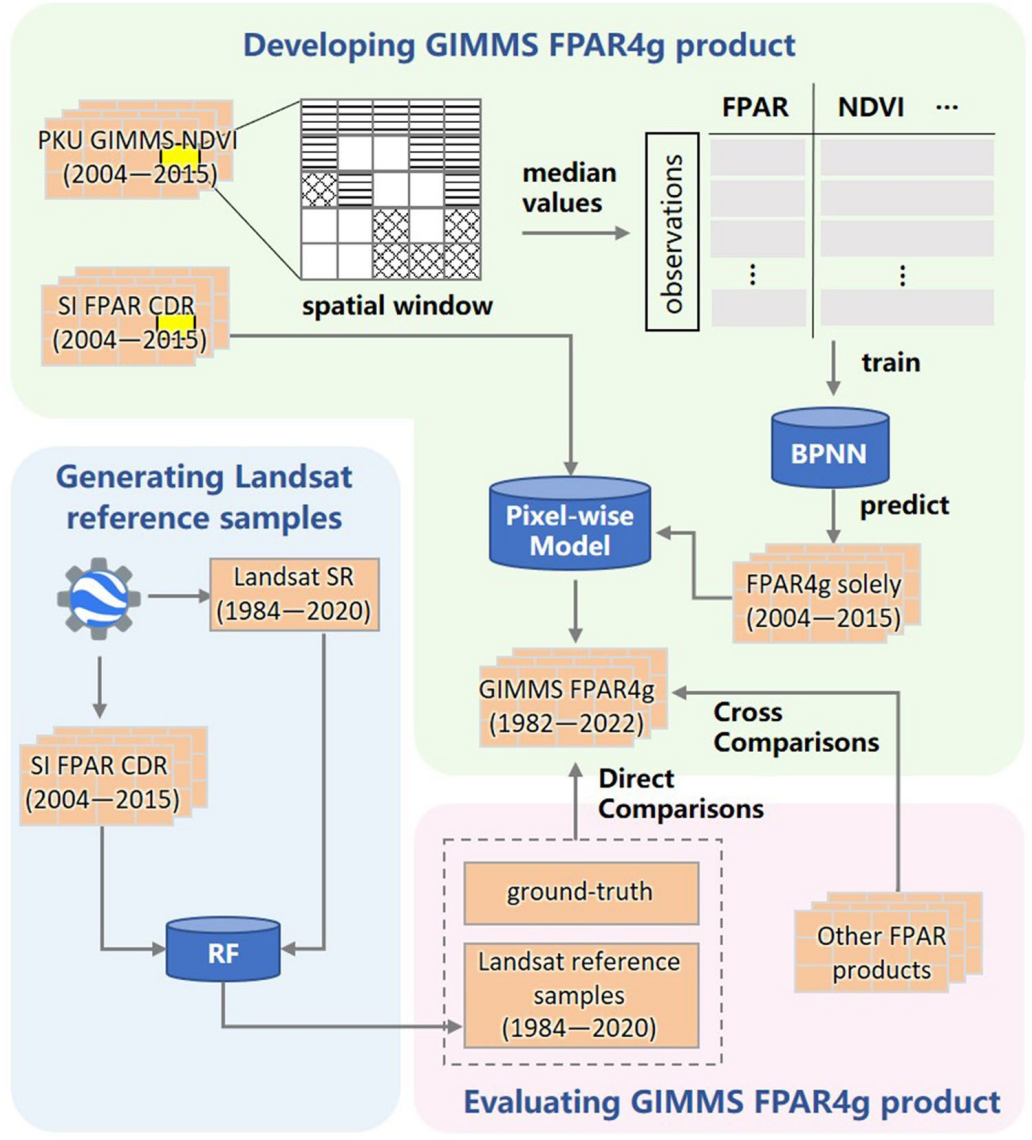
Schematic flowchart of the generation and evaluation of the GIMMS FPAR4g product.

The framework for generating and evaluating the GIMMS FPAR4g dataset is shown in Fig. 1, which consists of three major steps. First, we trained the BPNN models for each half-month using PKU GIMMS NDVI and SI FPAR to generate FPAR4g solely (1982–2015). Then, we extended the temporal span of the GIMMS FPAR4g (1982–2022) by integrating the SI FPAR and the FPAR4g solely using a pixel-wise regression model. Finally, the GIMMS FPAR4g was evaluated, including direct comparisons with ground-based measurements and Landsat reference samples, as well as inter-comparisons with other long-term global FPAR products. The details of each step were described in the following sections.

## Data Acquisition

### Remotely sensed data

We used four sets of global FPAR satellite-derived products (i.e., SI FPAR CDR, GIMMS FPAR3g, GLASS AVHRR FPAR, and Terrestrial CDR [TCDR]). The SI FPAR CDR served as the benchmark in this study^46^. The dataset demonstrates notable advantages: (1) Stringent filtering principles are applied to exclude poor-quality retrievals, ensuring the retention of high-quality samples and the reliability of the CDR. (2) By accounting for the effects of satellite transit times throughout the day, the composite dataset is independent of specific sensors and unaffected by individual single observation. (3) An advanced Spatial-Temporal Tensor completion model is employed^47^. It ensures high consistency between the filled data and the original high-quality data by considering correlations across spatial neighborhoods, interannual variations, and periodic characteristics. Validation with ground observations underscores the reliability of the SI FPAR CDR (R-squared (R^2^) = 0.79, Root Mean Square Error (RMSE) = 0.15), which exhibits greater stability and lower noise compared to sensor-dependent counterparts. The SI FPAR CDR includes versions with multiple temporal and spatial resolutions during 2000–2022. The 15-day 500 m version was used for generating Landsat reference samples, and it was aggregated to 1/12° for training the BPNN models. To reduce negatively biased noise in EBF, we applied the weighted Savitzky-Golay filter to the time series for each pixel^48,49^. Data points identified as negative outliers in the times series based on the three-sigma rule were assigned a weight of 0.1, while others were given a weight of 1. Due to the scarcity of high-quality observations in the early 2000s, we exclusively utilize SI FPAR data from the years 2004 to 2022 in this study.

The GIMMS FPAR3g, the GLASS AVHRR FPAR, and TCDR were used for cross-comparison with the GIMMS FPAR4g. The extended version of the GIMMS FPAR3g spans from 1982 to 2016 and features a temporal resolution of 15 days and a spatial resolution of 1/12°. This long-term global FPAR product is derived from the GIMMS NDVI3g and the improved MODIS FPAR dataset based on Artificial Neural Networks (ANN)^50^. We also collected the latest version of the GLASS AVHRR FPAR (http://www.glass.umd.edu/FAPAR/AVHRR/). This dataset’s spatial and temporal resolutions are 0.05° and 8 days, respectively, spanning from 1981 to 2018^51^. We resampled it into 1/12° using the bicubic resampling method, and composited it to half-month intervals based on the maximum value composite method. The TCDR, a 0.05° daily FPAR climate data record released by NOAA’s NCEI, covers the period from 1982 to the present^52^. This dataset is generated using ANN from LTDR AVHRR reflectance data (1982–2013) and VIIRS reflectance data (2014 to present). Given the substantial number of missing values in its spatial coverage, we conducted a preprocessing approach to ensure comparability with other FPAR products. Initially, we aggregated the data to 15-day intervals, then filled in the gaps with the Spatial-Temporal Tensor completion model^47^, and finally resampled it to a spatial resolution of 1/12°. The characteristics of all FPAR products used in this study are listed in Table 1.

**Table 1:** Satellite-derived FPAR products used in this study.

Product	Time Span	Spatial Resolution	Temporal Resolution	Data Source	Used Method
**GIMMS FPAR4g**	1982–2022	1/12°	15-day	PKU GIMMS NDVI	BPNN & pixel-wise model
**GIMMS FPAR3g**	1982–2016	1/12°	15-day	GIMMS NDVI3g	BPNN
**GLASS AVHRR FPAR**	1982–2018	0.05°	8-day	GLASS AVHRR LAI	vegetation structure method
**TCDR**	1982–2022	0.05°	daily	LTDR reflectance & VIIRS reflectance	ANN
**SI FPAR CDR**	2000–2022	500 m	15-day	MOD15A2H, MYD15A2H, VNP15A2H	ST-Tensor

PKU GIMMS NDVI is our main data source for developing GIMMS FPAR4g with global coverage. It is available in 1/12° spatial resolution for every half-month from 1982 to 2015 (https://zenodo.org/record/8253971). The quality control (QC) layer is inherited from the GIMMS NDVI3g^53^. This NDVI dataset was developed by machine learning algorithms using the extensive (3.6 million) cross-calibrated reference NDVI from Landsat observations as a bridge and MODIS NDVI as a benchmark. Compared to other previous NDVI datasets based on AVHRR, PKU GIMMS NDVI effectively tackled the issues related to the orbit drift of NOAA satellites and sensor degradation of AVHRR^45^.

We also obtained Landsat Collection 1 Tier 1 surface reflectance (SR) data from the Google Earth Engine (GEE) platform, including Landsat 5 (TM), Landsat 7 (ETM+), and 8 (OLI) with a spatial resolution of 30 m from 1984 to 2020. Each scene retains six spectral reflectance bands (Blue, Green, Red, Near-Infrared, Short-Wave Infrared 1, and Short-Wave Infrared 2) along with a quality assessment (QA) band. In the QA band, the CFmask algorithm identified saturated pixels as well as observations contaminated by clouds, cloud shadows, and snow as “bad quality”^54^. It also provides the spatial location, solar zenith, azimuth angle at the acquisition time, and atmospheric opacity (AOP).

### Ancillary data

We used the land cover map derived from MCD12Q1 with the Leaf Area Index (LAI) legacy classification scheme^55^, which provides yearly images from 2001 to 2020 at an original spatial resolution of 500 m. We focused on eight biome types: Grasslands (GRA), Shrublands (SHR), Broadleaf Croplands (CRO), Savannas (SAV), Evergreen Broadleaf Forests (EBF), Deciduous Broadleaf Forests (DBF), Evergreen Needleleaf Forests (ENF), and Deciduous Needleleaf Forests (DNF). We resampled them to 1/12° using the nearest-neighbor algorithm. Based on the biome types with the highest frequency of occurrence over 20 years, we generated the major biome type map for 1982–2022. The MCD12Q1 product cannot represent the biome types before 2000; however, its high compatibility with SI FPAR CDR minimizes potential discrepancies from integrating different data sources, making it the best available choice under current constraint.

### Ground-based data

Ground-based observations from four distinct sources were collected for a direct comparison with GIMMS FPAR4g. The Ground-Based Observations for Validation (GBOV) service offers *in-situ* datasets of ground-based monitoring sites (https://gbov.acri.fr). The GBOV Reference Measurements (RMs) database is created from raw measurements collected at sites that have undergone rigorous quality control procedures. Land Products (LP) are derived by upscaling the RMs. In this study, the GIMMS FPAR4g was evaluated using the LP product of FPAR (LP4)^56^. We conducted further processing and quality control of LP4 following the user support provided on the GBOV official website. Observations were averaged over a 3 × 3 km area. Firstly, LP values with input or output being out of range were masked before aggregated. Next, LP values obtained from high-resolution images acquired outside of the minimum and maximum day-of-year range of the calibration function were excluded. Finally, only LP values with more than 50% of valid native spatial resolution were aggregated. The DIRECT V2.1 database also supplies spatially-averaged FPAR values across a 3 × 3 km area^57^. Ground-based measurements are upscaled using high-spatial resolution imagery to reflect spatial heterogeneity. The dataset includes 176 global sites across 7 major biome types, spanning from 2000 to 2021 (https://calvalportal.ceos.org/lpv-direct-v2.1). Additionally, we have compiled ground reference data from the VALERI project during 2001–2005 (http://w3.avignon.inra.fr/valeri/) and the Implementing Multi-Scale Agricultural Indicators Exploiting Sentinels (ImagineS) project during 2013–2016 (http://fp7-imagines.eu/). Each VALERI site spans an area of 3 × 3 km, and each ImagineS site encompasses a 5 × 5 km area, rendering them appropriate for the validation of medium-resolution satellite-derived products. We aggregated measurements within identical spatial (1/12°) or temporal (half-month) frames, excluding reference data incongruent with our major biome types.

## Developing the GIMMS FPAR4g product

Numerous studies have demonstrated a strong correlation between NDVI and FPAR^58–60^. The relationship between them varies considerably across different temporal periods, geographical locations, and land covers^61–63^. ANNs are employed to deal with nonlinear relationships between independent and dependent variables with excellent predictive performance. The BPNN, one of the most popular and well-established ANN algorithms, has garnered extensive utilization in ecological research and the development of satellite-derived products^50,51,64^.

In this study, we employed a combination of the BPNN algorithm and a pixel-wise multi-sensor records integration approach to generate the GIMMS FPAR4g product. First, we obtain high-quality samples and train the BPNN models for each half-month. High-qualiy samples here refer to observations with a QC layer of 0 or 1 in the PKU GIMMS NDVI and their corresponding SI FPAR CDR. Specifically, we partitioned the globe into several N × N spatial windows and identified dominant biome types within each window as those covering more than 10% of the valid pixels. For each spatial window, the median values of SI FPAR and PKU GIMMS NDVI on each dominant biome were extracted. In the BPNN models, SI FPAR was designated as the target variable, while PKU GIMMS NDVI, the geographical coordinates of the spatial window’s center, and the biome type served as explanatory variables. The samples were divided into training, validation, and test data with proportions of 70%, 15%, and 15%. Bayesian optimization algorithms were used to determine the BPNN model’s optimal hyperparameters (i.e., learning rate and the number of hidden layer nodes) and the optimal spatial window size. Specifically, for EBF, a window size of 11 was used, while for other biome types, a window size of 25 was employed. The sample size and the optimal window size for each model are shown in Supplementary Table 1. We conducted 10 repetitions for each combination of parameters to reduce the uncertainty of BPNN model initialization. The average R^2^, RMSE, MAE, and MAPE values were used to evaluate each model’s performance on the test set (Supplementary Table 2). Then, the optimal model for each half-month was employed to generate FPAR4g solely (1982–2015).

Finally, we extended the temporal coverage of our GIMMS FPAR4g product (1982–2022) by referring to Mao *et al*.^65^. Specifically, we modeled regressions for each pixel based on the 15-day data of SI FPAR and FPAR4g solely during their overlapping period from 2004 to 2015. The Generalized Additive Model (GAM) is a flexible regression technique that allows for non-linear relationships between the dependent and independent variables. Therefore, GAM was used for pixels within EBF, and Ordinary Least Squares (OLS) regression was for the other biome types (Supplementary Fig. 1). We then applied the constructed pixel-wise regression model to the FPAR solely dataset predating 2004. The FPAR product, calibrated using the pixel-wise regression model, more closely approximates the SI FPAR, particularly in the EBF (Supplementary Figs 2–4).

## Generating Landsat FPAR reference samples

The Landsat mission is the longest-running Earth imaging program. Although long revisit cycles and cloudy scenes limit the global applications of the Landsat observations, their long continuity, stability, and radiometric and geometric accuracy enable long-term monitoring of changes^66,67^. To overcome the absence of ground observations before 2000 and validate the temporal consistency of the GIMMS FPAR4g and its performance over the entire period, we generated massive Landsat FPAR reference samples spanning 1984–2020. In this step, we established the relationship between SI FPAR CDR and Landsat data for their overlapping period (2004–2020), which ensures the consistency of Landsat data across different sensors and their comparability with SI FPAR CDR. Consequently, the Landsat reference samples are suitable for evaluating GIMMS FPAR4g.

### Training samples generation

High-quality sample pairs of Landsat SR and SI FPAR were extracted for each biome type. We screened out pixels (500 m) with constant biome type during 2004–2020 based on the land cover map. Using the systematic random sampling method, seventy thousand pure spatial sample locations were selected for DNF, and one hundred thousand for the remaining biome types. A matrix of 20 × 20 Landsat pixels (30 m) was extracted from Landsat 5, 7, and 8 during 2004–2020 with each spatial sample as the center (Supplementary Fig. 5).

We further purified the spatial samples by retaining only high-quality clear-sky Landsat observations and filtered the homogeneous spatial samples. The entire sample locations where the percentage of high-quality Landsat pixels was less than 90% or the average AOP exceeded the set threshold were excluded, details are given in^45^. Landsat data from the remaining spatial samples were aggregated to 500 m spatial resolution and automatically matched into sample pairs with the corresponding SI FPAR. The coefficient of variation (CV) can be expressed as the ratio of the mean and standard deviation of the Landsat SR corresponding to each SI FPAR pixel. We derived the CV for each spatial sample based on six spectral reflectance bands regarding Gao *et al*.^68^. Sample pairs with an average CV less than 0.15 across six bands were retained. We averaged the sample pairs with the same spatial location and acquisition time for subsequent modeling.

### Random Forest regressors construction

We trained a random forest (RF) regressor for each combination of biome and sensor (TM, ETM+, and OLI), taking into consideration variations in radiative transfer mechanisms across biomes and differences in spectral properties among sensors^69^. NDVI, the enhanced vegetation index (EVI), the two-band version of the enhanced vegetation index (EVI2), and the normalized difference water index (NDWI) can be derived from surface reflectance. Six spectral bands, four vegetation indices, latitude, longitude, solar zenith angle, and zenith angle at acquisition time were selected as explanatory variables, with corresponding SI FPAR as the response variable. We applied MATLAB 2022b implementation of RF using the function ‘TreeBagger’. Each regressor contained 200 decision trees, with the number of features to select for each split set to the default value (i.e., one-third of the number of features), and the minimum number of observations per leaf set to 5. R^2^, RMSE, Mean Absolute Error (MAE), and Mean Absolute Percentage Error (MAPE) were used to assess the performance of the model on the out-of-bag data. The performance of each RF regressor is shown in Supplementary Table 3.

### Landsat FPAR prediction

We acquired Landsat SR data for the years 1984 to 2020 via GEE as input data for the trained RF models and then generated Landsat FPAR reference samples. We randomly sampled 40,000 pixels (1/12°) from the major biome type map. Within each pixel, nine sample locations with a matrix of 20 × 20 Landsat pixels (30 m) were evenly distributed. Then, Landsat SR data for each sample location were extracted from all available scenes from Landsat 5, 7, and 8. In the same way as the quality control procedure described in *Training samples generation*, we filtered sample locations where the proportion of good quality pixels exceeded 90% and the AOP met the requirements we set. After purity screening, the Landsat SR data of the remaining sample locations was aggregated to a resolution of 500 m. FPAR predictions were obtained at each 500 m Landsat SR sample location based on the trained random forest regressors. If more than half of the sample locations (i.e., greater than 5) were available within each 1/12° pixel, the average of all valid predictions was performed. The reference samples were then aggregated at half-month, with samples falling within the same time domain being averaged. There were approximately 4.8 million high-quality Landsat reference samples for 1984–2020 (as shown in Supplementary Fig. 6). We attempted to validate the Landsat reference samples using ground-based measurements. However, due to discontinuities in the spatial and temporal distributions of these two datasets, the number of available samples was limited (Supplementary Fig. 7).

## Evaluating the GIMMS FPAR4g product

The evaluation of the GIMMS FPAR4g product includes direct comparisons with ground-based measurements and Landsat FPAR reference samples and inter-comparisons with other satellite-derived FPAR products.

### Direct validation

Direct validation of the GIMMS FPAR4g product involved two main components: validation with ground-based measurements and validation with Landsat FPAR reference samples.

First, we validated the GIMMS FPAR4g directly using four sets of collected ground-based measurements: DIRECT V2.1, GBOV, VALERI, and ImagineS. R^2^, RMSE, MAE, and MAPE were used as metrics to evaluate the accuracy of the GIMMS FPAR4g product. These datasets were generally collected after 2000.

We further independently evaluated the intraconsistency of each long-term global FPAR product (i.e., GIMMS FPAR4g, GIMMS FPAR3g, and GLASS AVHRR FPAR) against the massive Landsat FPAR reference samples spanning 1984–2020 generated by Section **Generating Landsat FPAR reference samples**. The bias between the Landsat reference data and the FPAR product can be used to measure the magnitude of orbital drift and sensor degradation effects. We de-seasonalized the time series of bias for each scene and extracted interannual nonlinear trends for each NOAA satellite mission using the Ensemble Empirical Mode Decomposition (EEMD). The EEMD algorithm was done in MATLAB 2022b. We set the noise level to 0.2, ensemble number to 100, and number of prescribed intrinsic mode function to 8.

Finally, the terrestrial vegetation area was divided into 2° × 2° grids, then we calculated the MAE, RMSE, and correlation coefficient (R) between the Landsat reference data and each set of satellite-derived products within each grid cell. Note that in the aforementioned direct comparisons, we analyzed their common valid samples to all four datasets (three long-term FPAR products and Landsat reference data).

### Indirect validation

We assessed the interconsistency of GIMMS FPAR4g with other global FPAR products (GIMMS FPAR3g, GLASS AVHRR FPAR, and SI FPAR CDR). These products were resampled to a half-month temporal resolution and a 1/12° spatial resolution prior to comparison. For each product, pixels with FPAR values below 0.05 were excluded. The annual mean FPAR was calculated by averaging the FPAR values across 24 scenes throughout the year. The global mean FPAR was derived from an area-weighted average of the annual mean FPAR of terrestrial vegetation regions.

Initially, we calculated the time series of mean FPAR values across different latitudinal bands from 1982 to 2016 to qualitatively evaluate the differences among the three long-term FPAR products. Then, we calculated the linear trends for pre- and post-2000 periods for the four products over their respective available periods, and detected decadal-scale nonlinear trends following Jiang *et al*.^70^. Specifically, we decomposed three long-term FPAR products into four components and the SI FPAR into three components using EEMD, subsequently summing the last two components of each product. Additionally, we compared annual linear trends and interannual changes across the FPAR products. The overlapping period of these products was divided into three intervals: 1982–2016 (p1), 1982–2003 (p2), and 2004–2016 (p3). For each period, we calculated linear trends for the entire globe and eight biomes and assessed their statistical significance using the Mann-Kendall test. Finally, we examined the spatial patterns of annual linear trends as well as interannual variability (IAV) for each set of products over time. We detrended the time series on each pixel and used the coefficient of variation (CV), defined as the ratio of the standard deviation to the mean, to indicate the IAV of FPAR. Quantifying IAV using the CV eliminates differences in magnitude across products, ensuring comparability of results.

## Data Records

This dataset and its Readme file are available from Zenodo^71^. The GIMMS FPAR4g dataset offers a temporal resolution of half a month and a spatial resolution of 1/12°, spanning from 1982 to 2022. We present the linear trend of GIMMS FPAR4g across three distinct time periods: 1982–1999, 2000–2022, and 1982–2022, as well as the results of the Mann-Kendall statistical significance test (Supplementary Fig. 8). We have created a separate file in GeoTIFF format for each scene. There are 24 scenes for each year, each of which represents FPAR for half a month. Each scene is associated with a quality control layer inherited from PKU GIMMS NDVI (version: solely). Additionally, we provide global images showcasing the correlation coefficient and RMSE of the pixel-wise regression models for data users. The FPAR4g solely dataset (1982–2015) which has not been calibrated with SI FPAR is also stored in the same repository. It is strongly recommended that users of the GIMMS FPAR4g product read the Readme file before use to ensure proper handling of the fill values and QC flags within the dataset.

## Technical Validation

### Direct validation with ground-based measurements and Landsat FPAR reference samples

**Fig. 2:**
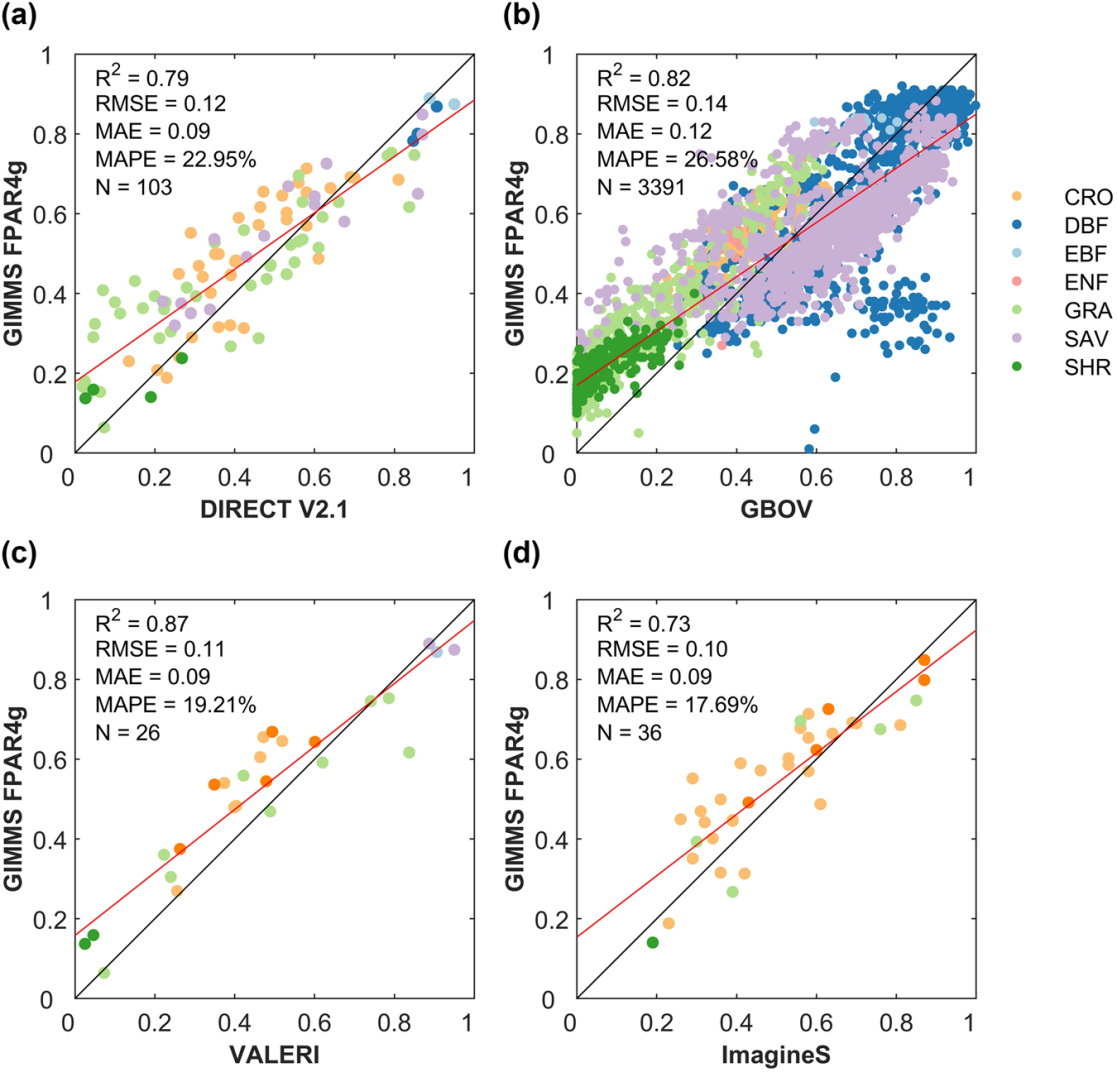
Validation of GIMMS FPAR4g with ground-based measurements. (**a**) DIRECT V2.1 database, (**b**) GBOV, (**c**) VALERI, and (**d**) ImagineS.

Four independent sets of ground-based measurements were used for direct validation of the GIMMS FPAR4g product. As shown in Fig. 2, the GIMMS FPAR4g dataset developed in this study is in good agreement with two independent ground-based measurements across the entire FPAR range (R^2^ = 0.73–0.87, RMSE = 0.10–0.14, MAE = 0.09–0.12, MAPE = 17.69%-26.58%). GIMMS FPAR4g shows a systematic overestimation for low FPAR values in *in-situ* observation datasets, while it displays a systematic underestimation for high FPAR values. Similar issues are observed in the SI FPAR dataset that we used as the target variable for the BPNN models^46^. Overall, our product demonstrates good consistency with ground reference data post-2000, with the RMSE closely approaching the GCOS accuracy requirement for FPAR (10%).

**Fig. 3:**
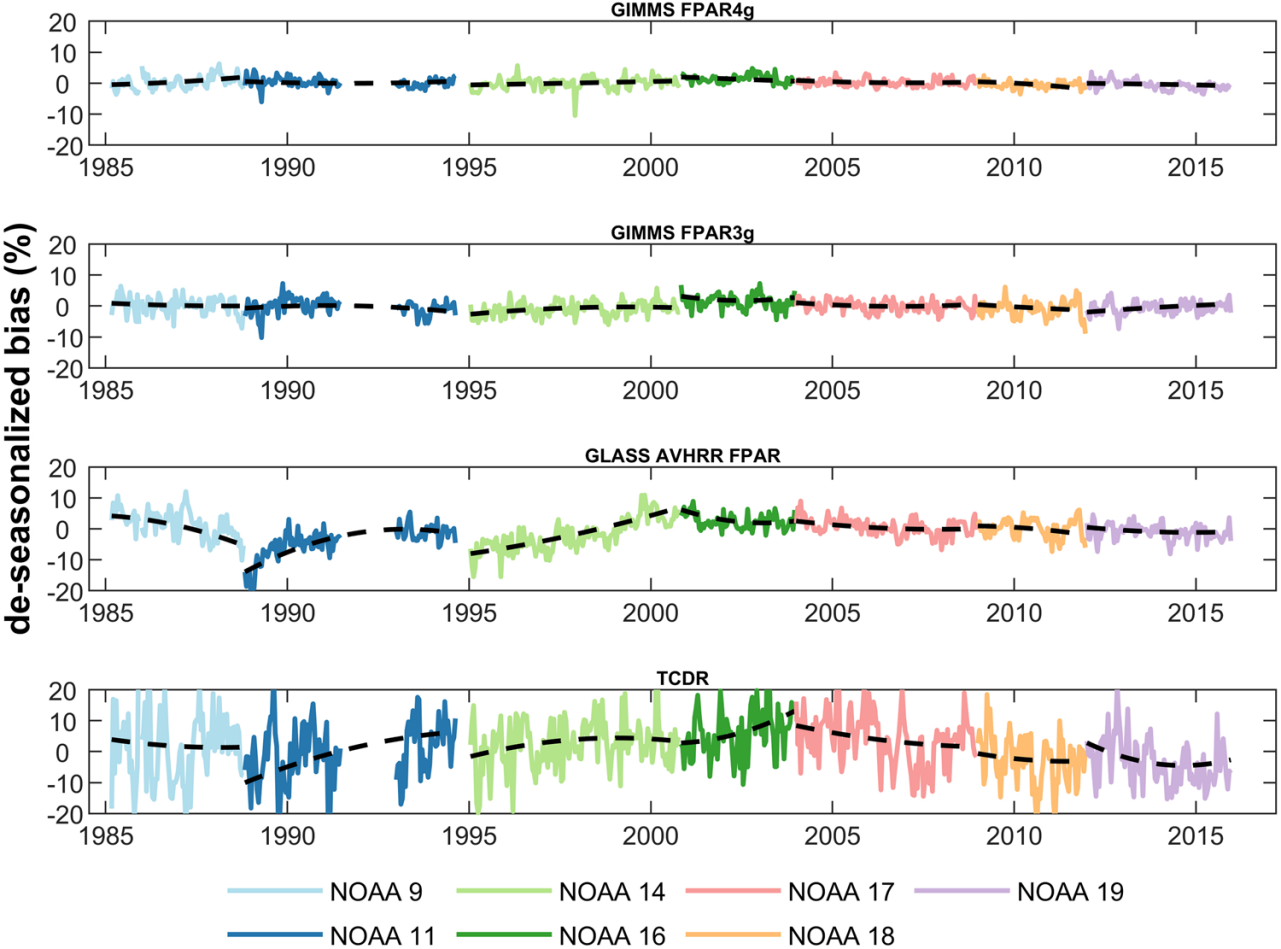
Temporal variations of de-seasonalized bias of four products on a global scale. Values from seven NOAA satellite missions are shown in different colors. Each black dashed line represents the nonlinear trend extracted using the EEMD method during the corresponding NOAA satellite mission.

We evaluated the temporal consistency of the GIMMS FPAR4g and other two FPAR products using Landsat reference data as a benchmark. The more pronounced the fluctuations in deseasonalized bias, the greater the intraconsistency of the time series. The abrupt changes observed between different NOAA satellite missions reflect the impact of satellite platform transitions on the temporal consistency of the data. Additionally, the nonlinear trends of the bias extracted using EEMD are utilized to indicate the severity of the effects of orbital drift and sensor degradation. As shown in Fig. 3, the bias between GIMMS FPAR4g and Landsat samples does not deviate from the reference values with increasing NOAA launch years and always remains within ±10%. In contrast, GLASS AVHRR FPAR and TCDR show significant nonlinearity in trends, implying severe effects of orbital drift and sensor degradation. We also found far more dramatic bias fluctuations in the remaining three products than those of GIMMS FPAR4g, as well as more severe jumps when NOAA satellite platforms are changed. This discrepancy arises because challenges like satellite platform replacement, sensor degradation, and orbital drift have not been adequately addressed in the data sources for GIMMS FPAR3g, GLASS AVHRR FPAR, and TCDR inversions. However, the development of the GIMMS FPAR4g leverages the PKU GIMMS NDVI, for which issues related to orbital drift and sensor degradation have been effectively addressed and rectified. Similar to the global scale findings, our product exhibited notable temporal consistency within each biome (Fig. S9-S16).

**Fig. 4:**
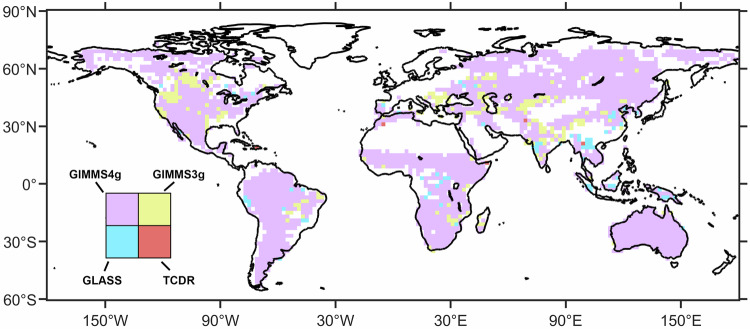
Geographic distribution of FPAR products with the smallest MAE with the Landsat FPAR reference samples among four products. GIMMS FPAR4g (GIMMS4g for short), GIMMS FPAR3g (GIMMS3g for short), GLASS AVHRR FPAR (GLASS for short).

We then assessed the performance of three products over the entire period. As shown in Fig. 4, the color of each 2° × 2° grid represents the FPAR product that exhibits the smallest error in comparison to the Landsat reference samples. To mitigate the effect of sample size on accuracy assessment, we omitted grids containing fewer than 100 samples. Most global regions are depicted in purple, indicating that GIMMS FPAR4g has a predominant advantage over the four products. Similar conclusions can be drawn using RMSE and correlation coefficient as evaluation metrics (Supplementary Fig. 17).

### Indirect validation with other FPAR products

**Fig. 5:**
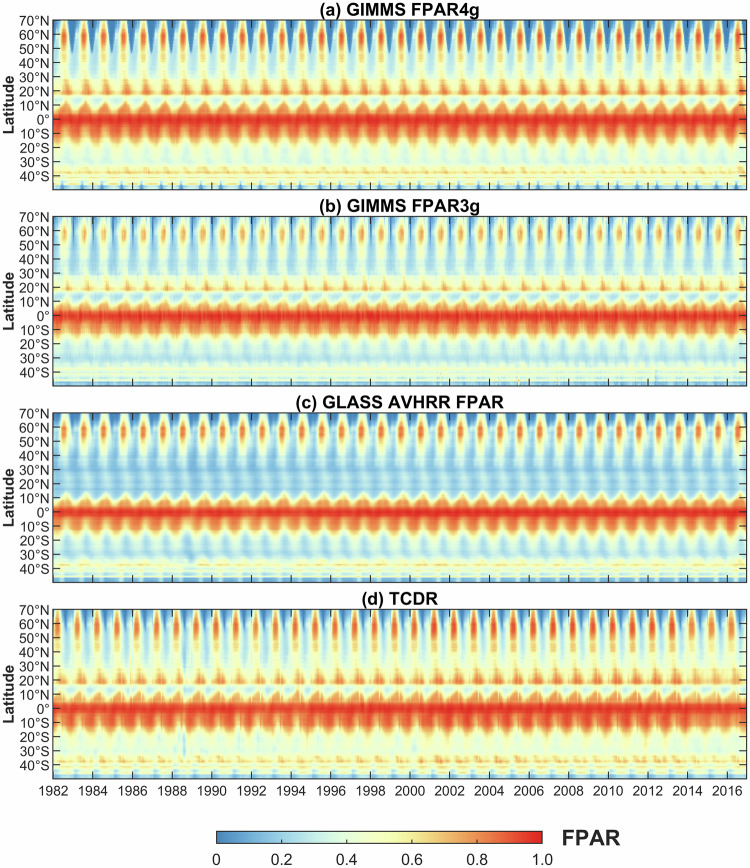
Hovmöller diagrams of the mean values of the (**a**) GIMMS FPAR4g, (**b**) GIMMS FPAR3g, (**c**) GLASS AVHRR FPAR, and (**d**) TCDR for latitudinal bands during 1982–2016.

Hovmöller diagrams were used to conduct a qualitative analysis of four long-term FPAR products across latitudinal bands over time (Fig. 5). These products consistently demonstrate divergent seasonal FPAR patterns in the Northern and Southern Hemisphere. Notably, the highest mean FPAR values are observed in the equatorial regions, accompanied by a secondary peak during the growing season in northern-high-latitude areas. The primary distinction among these products is most apparent between 20°N and 30°N. In this region, the mean values of GIMMS FPAR4g, GIMMS FPAR3g, and TCDR are depicted as yellow to red during the growing season, whereas those of GLASS AVHRR FPAR are lower and appear blue. Overall, the GIMMS FPAR4g shows the most similar pattern to that of the TCDR from 1982 to 2016.

**Fig. 6:**
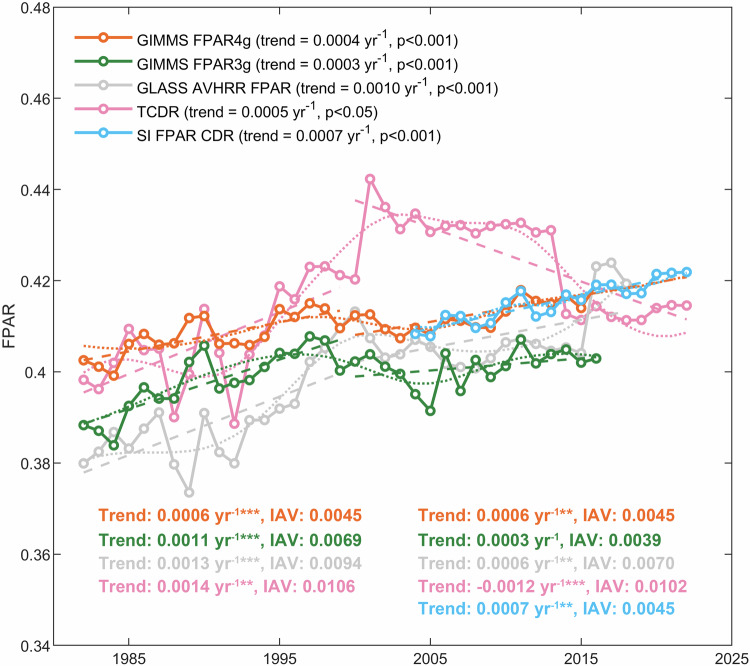
Global annual FPAR variations for five products. The dotted lines indicate the decadal-scale trends detected by the EEMD method. The linear trends of the three long-term products for the pre- and post- 2000 periods and the SI FPAR for 2004–2022 are shown as dashed lines (Mann-Kendall test, *p < 0.05, **p < 0.01, ***p < 0.001). Interannual variability (IAV) is calculated as the standard deviation of the global annual anomalies of FPAR over its individual period.

All FPAR products exhibit significant linear increases over their respective available periods (ranging from 0.0003 to 0.0010 yr^−1^, p < 0.001; Fig. 6). Prior to 2000 (1982–1999), the TCDR show the most significant upward trend (0.0014 yr^−1^, p < 0.05), followed by the GLASS AVHRR FPAR (0.0013 yr^−1^, p < 0.001) and then by the GIMMS FPAR3g (0.0011 yr^−1^, p < 0.001) and the GIMMS FPAR4g (0.0006 yr^−1^, p < 0.001). After entering the year 2000, the GIMMS FPAR4g maintains a notable increasing trend, whereas the upward trends of the other three long-term FPAR products slow down. TCDR is the only product with a significant linear downward trend in the post-2000 period (−0.0012 yr^−1^, p < 0.001). In addition, our product displays consistent interannual variability across both individual periods (1982–1999: 0.0045, 2000–2022: 0.0045), contrasting with the GIMMS FPAR3g and the GLASS AVHRR FPAR, both of which exhibit reduced interannual variability after 2000 (GIMMS FPAR3g: from 0.0069 to 0.0039; GLASS AVHRR FPAR: from 0.0094 to 0.0070). Notably, our product closely aligns with the high-quality SI FPAR, emphasizing its reliability and accuracy.

Meanwhile, the dotted lines illustrated in Fig. 6 elucidate substantial variations among these products concerning decadal-scale nonlinear trends. During the initial decade (1982–1991), the GLASS AVHRR FPAR and the GIMMS FPAR4g both show a flat tendency, while the GIMMS FPAR3g displays an upward trajectory. TCDR undergoes drastic fluctuations with a marginal downward trend during this period. The subsequent decade (1992–2001) witnesses both GIMMS FPAR4g and GIMMS FPAR3g initially rising and then experiencing slight declines, while the GLASS AVHRR FPAR and TCDR notably increase. In the third decade (2002–2011), the GIMMS FPAR4g and the GIMMS FPAR3g remain relatively consistent with a minor upward trend, whereas the GLASS AVHRR FPAR product initially declines, then rises. TCDR, however, distinctly diverges by maintaining stability initially and then undergoing a sharp decline in 2013, highlighting its unique trend among the products examined. These abrupt changes may not represent actual signials but are likely attributed to shifts in data sources. Specifically, GLASS AVHRR FPAR was derived from LTDR AVHRR reflectance before 2000 and from MODIS surface reflectance after 2000. Similarly, TCDR transitioned from using AVHRR reflectance to VIIRS after 2013.

**Fig. 7:**
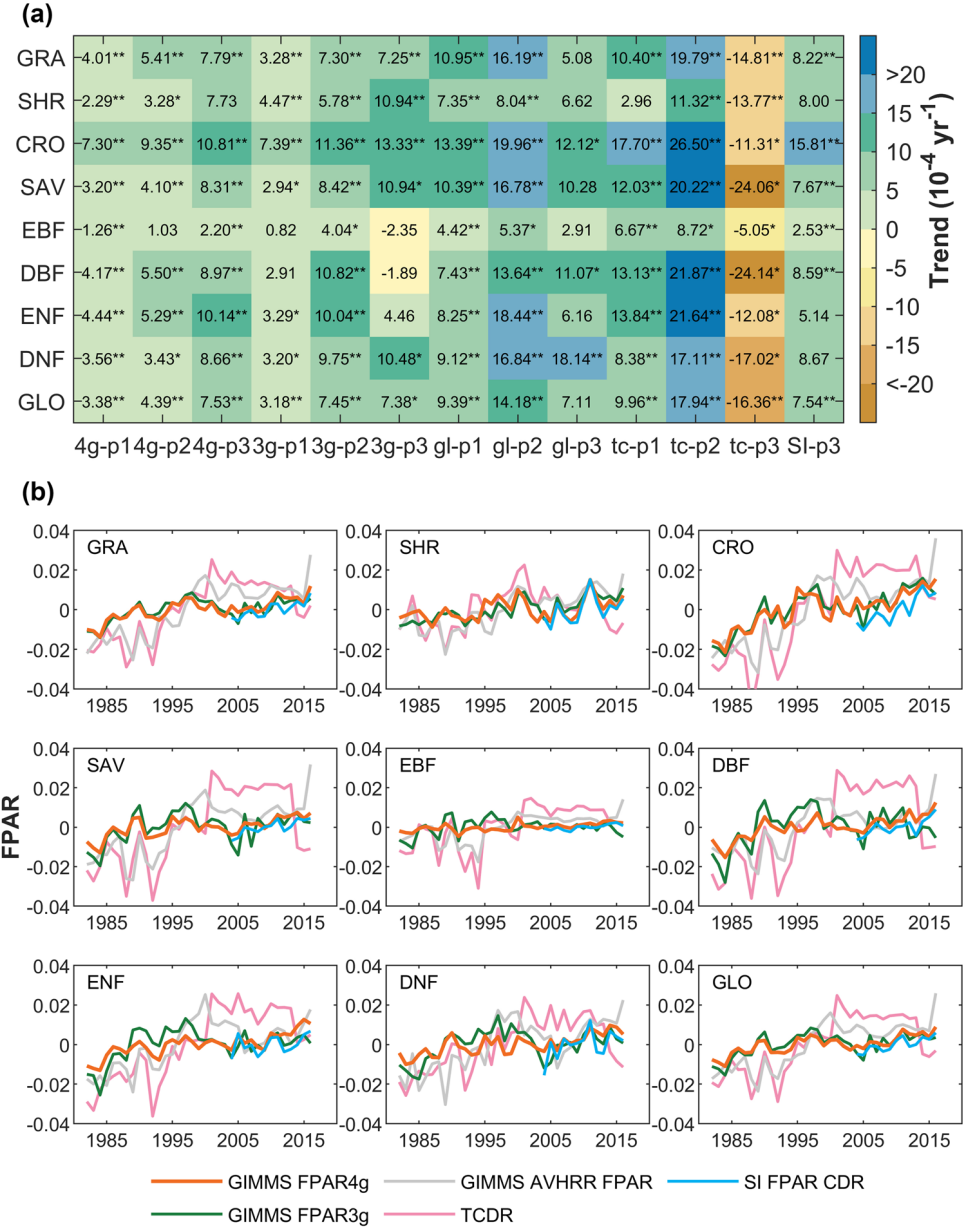
Trends and annual anomalies in FPAR for four products. (**a**) Trends in four products for each biome type. The period of this study is divided into 1982–2016 (p1), 1982–2003 (p2), and 2004–2016 (p3). The abbreviations 4g, 3g,gl, tc, and SI correspond to the five products: GIMMS FPAR4g, GIMMS FPAR3g, GLASS AVHRR FPAR, TCDR, and SI FPAR CDR, respectively. GLO represents trends on a global scale. In heatmap, values marked with ‘*’ represent p < 0.05 for trends under the Mann-Kendall test, while values marked with ‘**’ represent p < 0.01. (**b**) Interannual changes in anomalies of five products.

The annual mean FPAR anomalies across all datasets reveal similar conclusions for each biome type, as illustrated in Fig. 7b. The trends in the GIMMS FPAR3g, the GLASS AVHRR FPAR, and TCDR show marked changes around the year 2000. Specifically, these products transition from a significant upward trend to a plateau or a decreasing trend. Our product demonstrates more uniform interannual variability and trends over the entire period examined. On almost all biome types, the TCDR exhibits the most pronounced fluctuations, followed by the GLASS AVHRR FPAR, then the GIMMS FPAR3g, and the GIMMS FPAR4g.

We further quantitatively compared the annual linear trends of each product over different periods. The linear trends of SI FPAR can only be calculated in period 3. The most significant upward and downward trends are observed in the TCDR. Apart from the TCDR, the remaining four products generally show an upward trend in most biomes during each evaluated period. However, there are several instances of declining FPAR trends observed in the EBF and DBF during period 3 of the GIMMS FPAR3g (Fig. 7a). Across the three evaluated periods, the GIMMS FPAR4g shows its most pronounced increase during period 3, while the GIMMS FPAR3g, the GLASS AVHRR FPAR, and TCDR are in period 2. The trends in 4g-p3 and SI-p3 are close across all biome types and on a global scale.

**Fig. 8:**
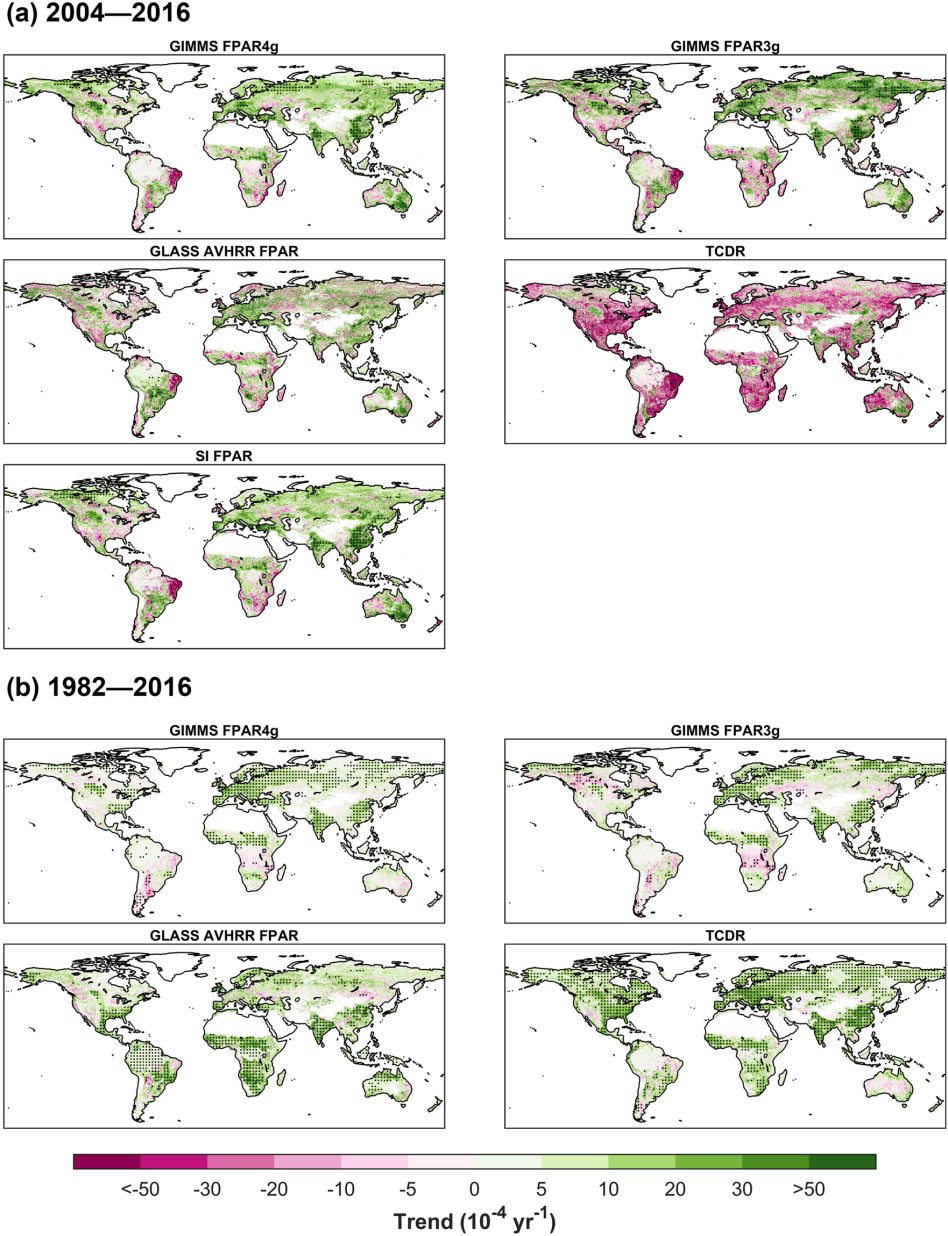
Comparison of spatial patterns of annual linear trends in FPAR products. (**a**) represent annual trends for GIMMS FPAR4g, GIMMS FPAR3g, GLASS AVHRR FPAR, TCDR, and SI FPAR CDR during 2004–2016. (**b**) represent annual trends for GIMMS FPAR4g, GIMMS FPAR3g, GLASS AVHRR FPAR, and TCDR during 1982–2016. Regions marked with dots indicate their trends are statistically significant (Mann-Kendall test; p < 0.05).

The geographic distributions of annual trends vary considerably both among various products and across different periods within the same product. Figure 8a displays the spatial patterns of trends in all products spanning the years 2004–2016. The trend in TCDR differs from all other products, displaying a decreasing trend across the vast majority of the globe. Among the four FPAR long-term products, the linear trends of the GIMMS FPAR4g and the SI FPAR are the closest, both indicating widespread darkening in China, India, Europe, and the northern-high-latitudes regions. In contrast, the GLASS AVHRR FPAR and the GIMMS FPAR3g show more significant differences when compared to the SI FPAR (Supplementary Fig. 18a). In the northern high latitudes, the GIMMS FPAR3g overestimates the darkening trend relative to the SI FPAR, while the GLASS AVHRR FPAR underestimates it. Meanwhile, the GIMMS FPAR3g accurately captures the remarkable darkening trends in India and China over this period, a phenomenon that the GLASS AVHRR FPAR fails to reveal.

Between 1982–2016, the areas experiencing significant darkening were considerably more extensive compared to the period from 2004–2016 (Fig. 8a,b). During this period, these products all captured the notable increasing trend in FPAR over 35 years in regions including China, India, Europe, and the Sahel region. Among them, the TCDR shows the most extensive range of darkening. Throughout the examined period, the linear trends of the GIMMS FPAR3g and the GIMMS FPAR4g exhibit minimal divergence across most areas. In northern North America, south of the Congo Basin, and the Central-Asia arid region, the GIMMS FPAR3g demonstrates more pronounced browning trends compared to our product. Conversely, the GIMMS FPAR3g records more significant darkening in eastern Australia, southwestern South America, and the northern-high-latitudes regions (Supplementary Fig. 18b). Meanwhile, the GLASS AVHRR FPAR identifies considerable darkening trends across vast expanses of South America, Western Australia, and nearly the entirety of Africa, which are not reflected in the other three datasets.

We then investigated the spatial distribution of IAV for each FPAR product (Supplementary Fig. 19). For both periods (1982–2003 and 2004–2016), the IAV of GLASS AVHRR FPAR was generally higher than that of other products, particularly in northern-high-latitude areas, Australia, and southern Africa. During 2004–2016, the spatial patterns of IAV in GIMMS FPAR4g and TCDR are similar to those of SI FPAR CDR, while GIMMS FPAR3g shows slightly higher IAV in northern-high-latitude areas compared to the other three products. The spatial patterns of IAV for GIMMS FPAR4g, GIMMS FPAR3g, and TCDR are generally consistent across both periods. We observed greater fluctuations in arid and semi-arid regions such as Australia, the southwestern United States, southern Africa, and Central Asia, and smaller fluctuations in regions like the Amazon, Congo Basin, and tropical rainforests in Southeast Asia.

Overall, the GIMMS FPAR4g product offers an extensive global FPAR record spanning 41 years, successfully meeting the GCOS ideal target for temporal span. With its 15-day temporal resolution, it closely approximates the temporal intervals stipulated by the GCOS. Comparisons with multiple sets of ground-based observations demonstrate that our product’s accuracy approaches the threshold specified by the GCOS. The most significant advancement in our work is addressing the temporal inconsistencies in long-term FPAR products attributed to the limitations of the AVHRR series. Our GIMMS FPAR4g product is expected to be a valuable asset in accurately monitoring global vegetation dynamics.

### Supplementary information

Supplementary Information

## Data Availability

The MATLAB codes for generating the GIMMS FPAR4g product are available on GitHub at https://github.com/WeiqingZhao/GIMMS_FPAR4g.
