# Supplementary material for: A global dataset of the fraction of absorbed photosynthetically active radiation for 1982–2022
Source: Sci Data. 2024 Jun 28;11:707. doi: 10.1038/s41597-024-03561-0 (PMC11213951; doi:10.1038/s41597-024-03561-0)
Supplement: Supplementary file 1 — Supplementary Information [file 41597_2024_3561_MOESM1_ESM.pdf]

A global dataset of the fraction of absorbed photosynthetically active radiation for 1982—2022

Supplementary Information

Table of Contents

Fig. S1 2
Fig. S2 3
Fig. S3 4
Fig. S4 5
Fig. S5 6
Fig. S6 7
Fig. S7 8
Fig. S8 9
Fig. S9 10
Fig. S10 11
Fig. S11 12
Fig. S12 13
Fig. S13 14
Fig. S14 15
Fig. S15 16
Fig. S16 17
Fig. S17 18
Fig. S18 19
Fig. S19 20
Table S1. 21
Table S2. 22
Table S3. 23

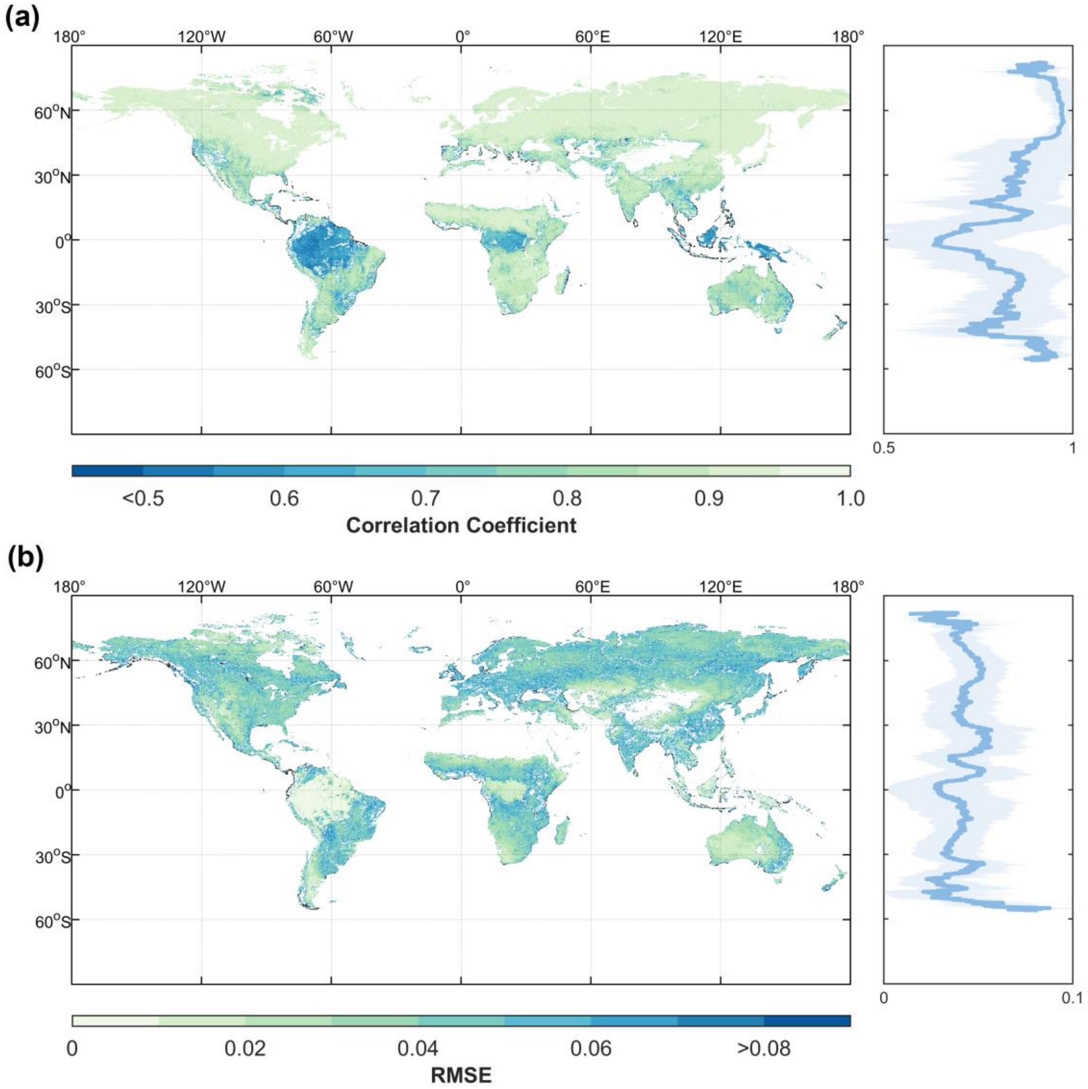

Fig. S1 The geographic distribution of the (a) correlation coefficients and (b) root mean square error (RMSE) for pixel-wise regression models.

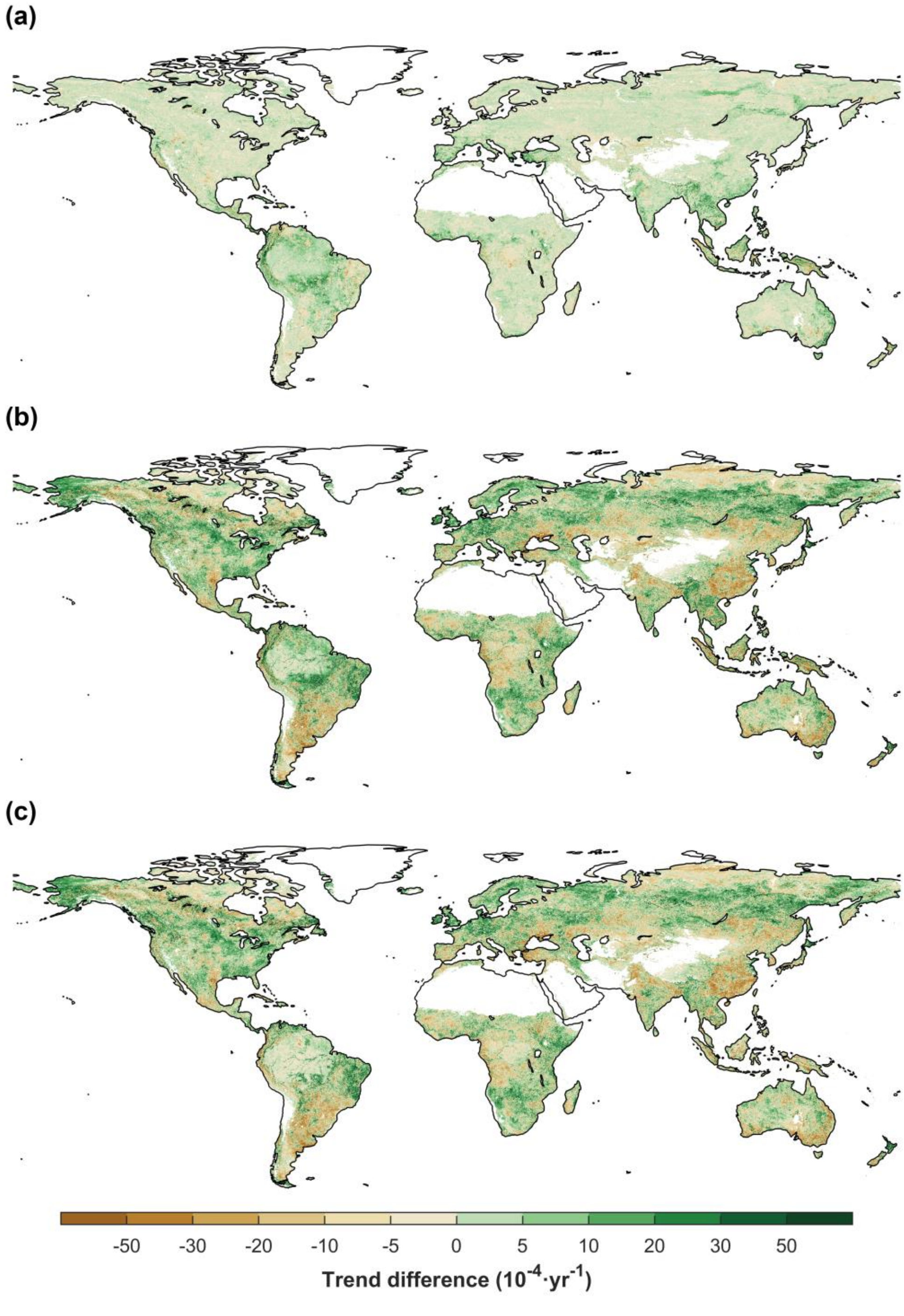

Fig. S2 Comparison of the global FPAR annual linear trends among three FPAR products: FPAR4g solely, the GIMMS FPAR4g, and SI FPAR CDR. (a) the trend difference between FPAR4g solely and GIMMS FPAR4g, (b) the trend difference between FPAR4g solely and SI FPAR CDR, and (c) the trend difference between GIMMS FPAR4g and SI FPAR CDR.

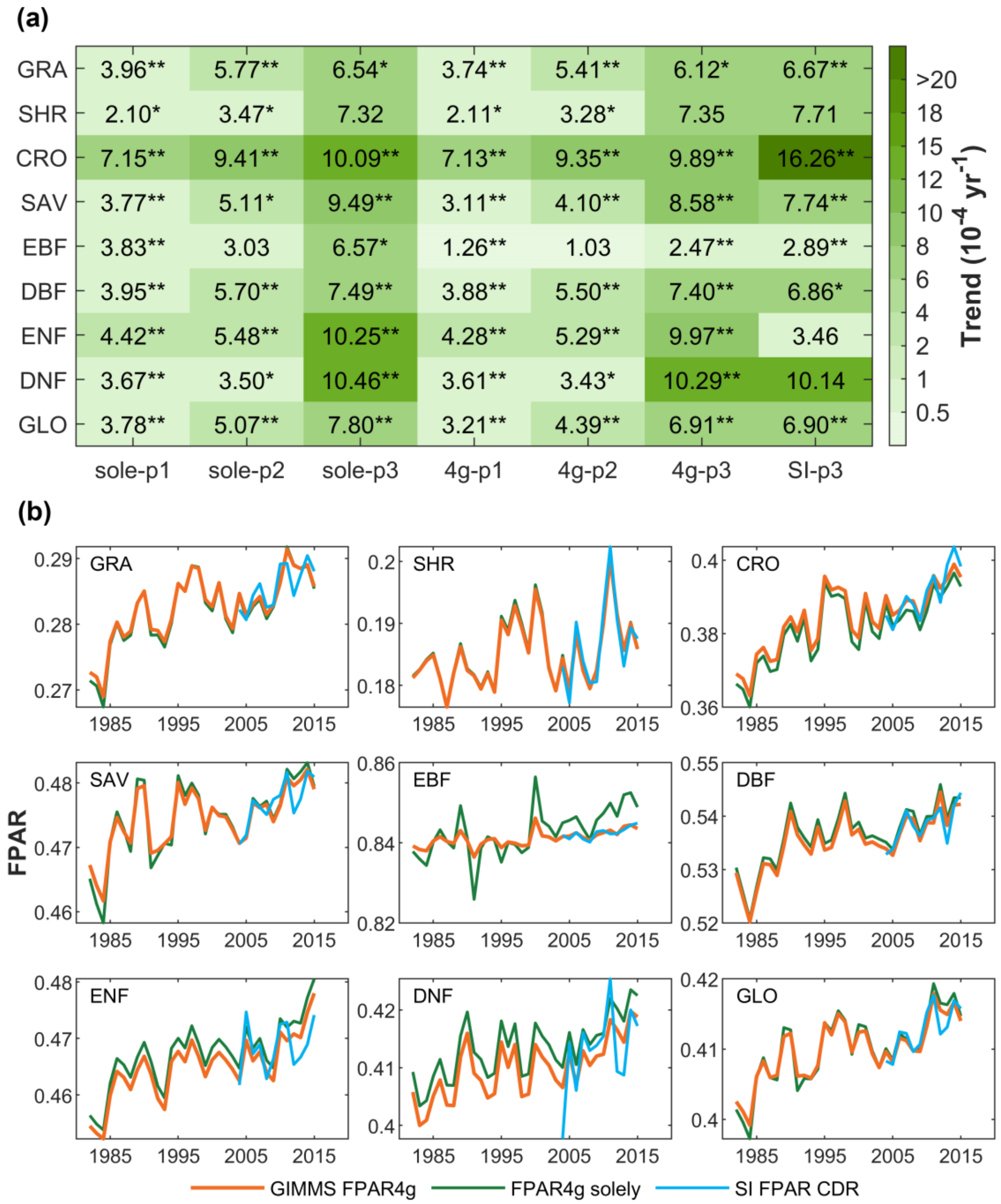

Fig. S3 Trends and annual anomalies in FPAR for three products. (a) Trends in three products for each biome type. The period of this study is divided into 1982—2015 (p1), 1982—2003 (p2), and 2004—2015 (p3). The abbreviations sole, 4g, and SI correspond to the three products: FPAR4g solely, GIMMS FPAR4g, and SI FPAR CDR, respectively. GLO represents trends on global scale (Mann-Kendall test; *p<0.05, **p<0.01). (b) Interannual changes in anomalies of four products.

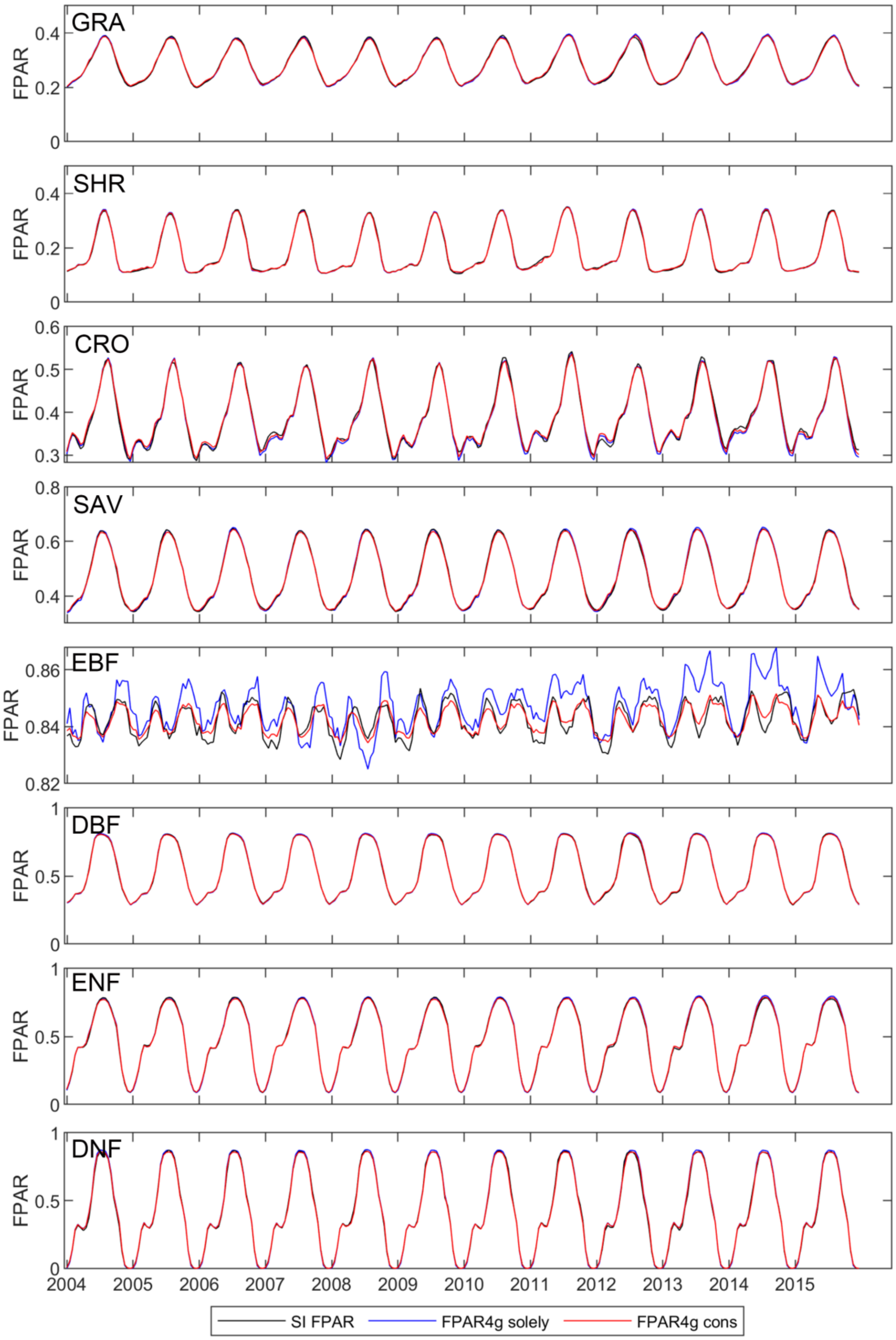

Fig. S4 Interannual variability of the mean values of the GIMMS FPAR4g, FPAR4g solely, and SI FPAR CDR for different biome types during 1982-2015.

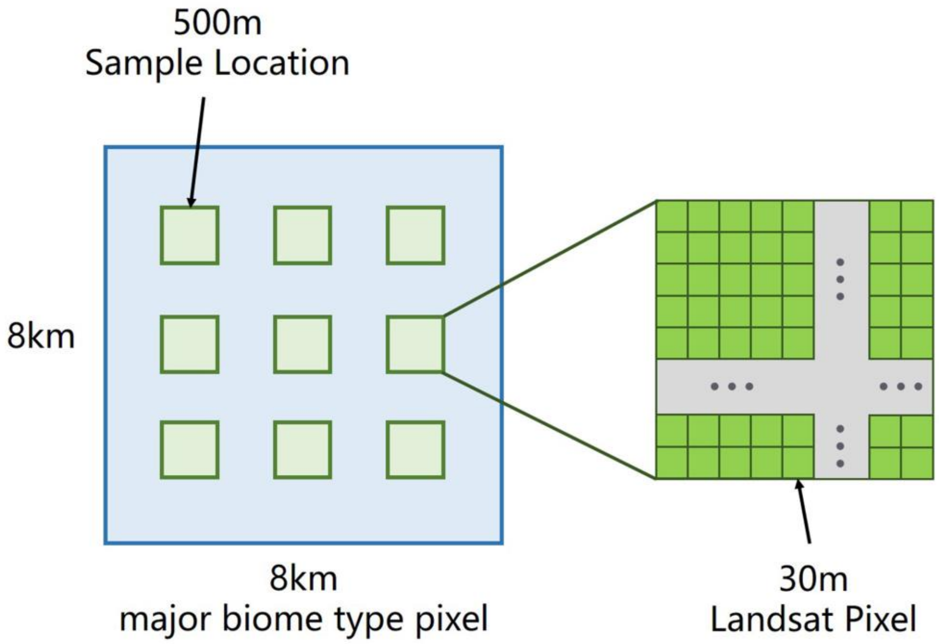

Fig. S5 Secondary sampling scheme for Landsat surface reflectance.

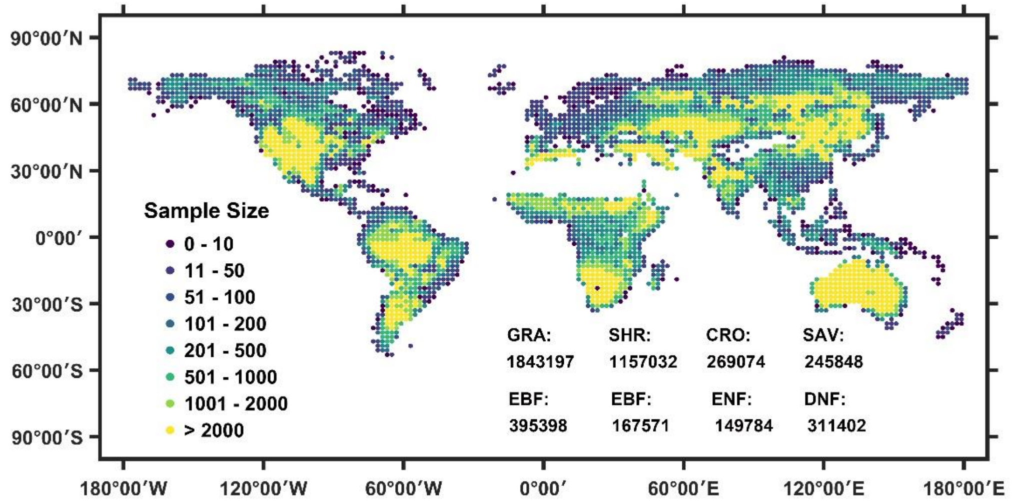

Fig. S6 The geographic distribution of the Landsat FPAR reference samples in 2° grid. GRA: Grasslands; SHR: Shrublands; CRO: Broadleaf Croplands; SAV: Savannas; EBF: Evergreen Broadleaf Forests; DBF: Deciduous Broadleaf Forests; ENF: Evergreen Needleleaf Forests; DNF: Deciduous Needleleaf Forests.

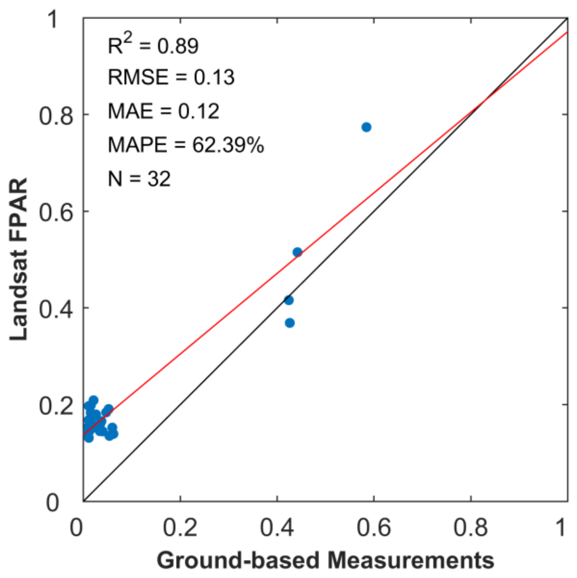

Fig. S7 Validation of Landsat FPAR reference samples with ground-based measurements from DIRECT V2.1 database, GBOV, VALERI, and ImagineS.

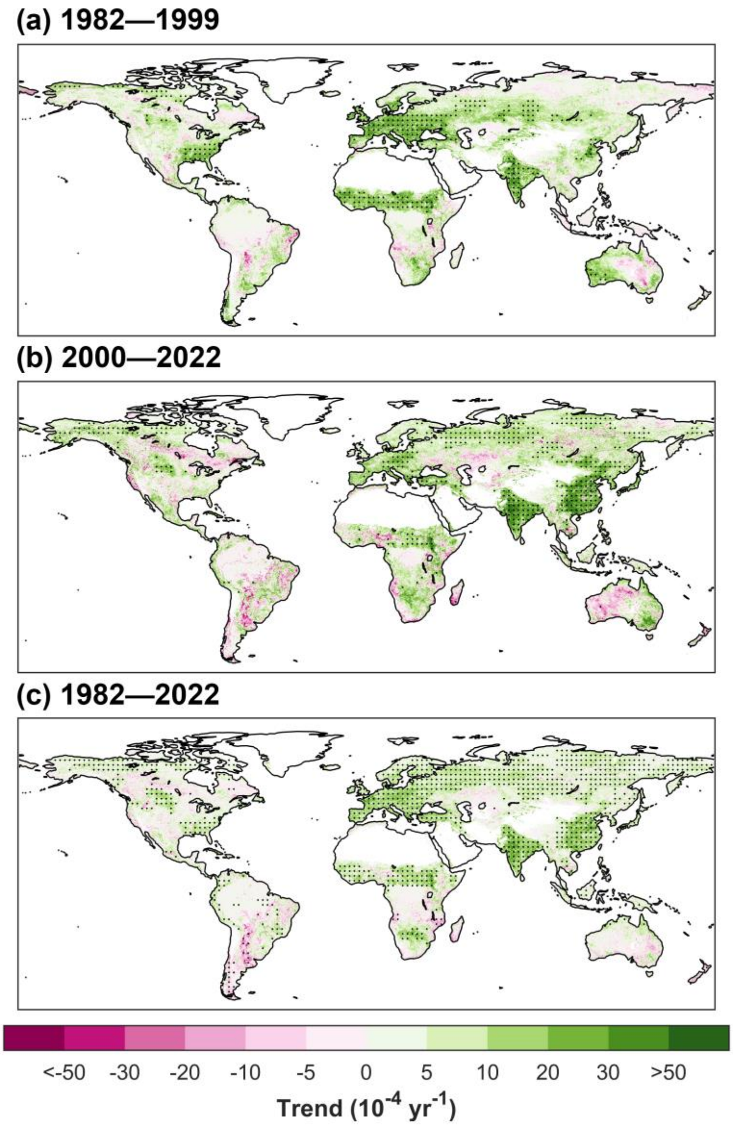

Fig. S8 Spatial distribution of the annual linear trends in GIMMS FPAR4g for (a) 1982—1999, (b) 2000—2022, and (c) 1982—2022. Regions marked with dots indicate their trends are statistically significant (Mann-Kendall test; $p<0.05$).

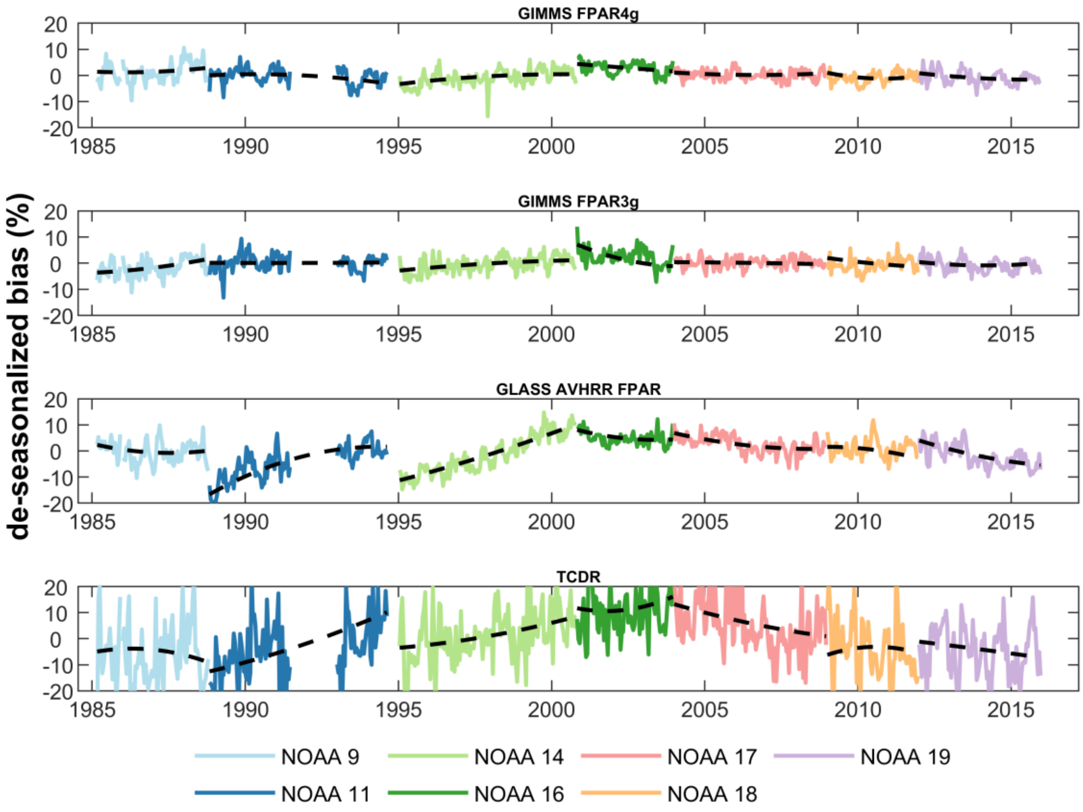

Fig. S9 Temporal variations of de-seasonalized bias of four products in GRA. Values from seven NOAA satellite missions are shown in different colors. Each black dashed line represents the nonlinear trend extracted using the EEMD method during the corresponding NOAA satellite mission.

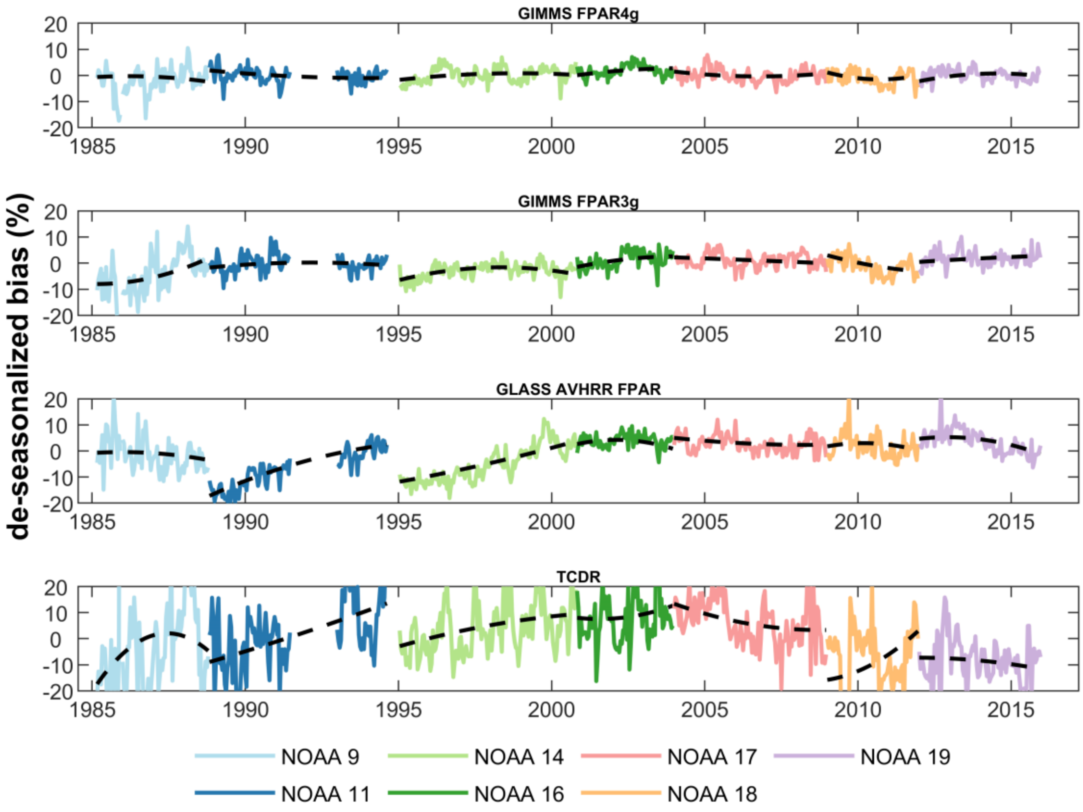

Fig. S10 Same as the Figure S9 but in SHR.

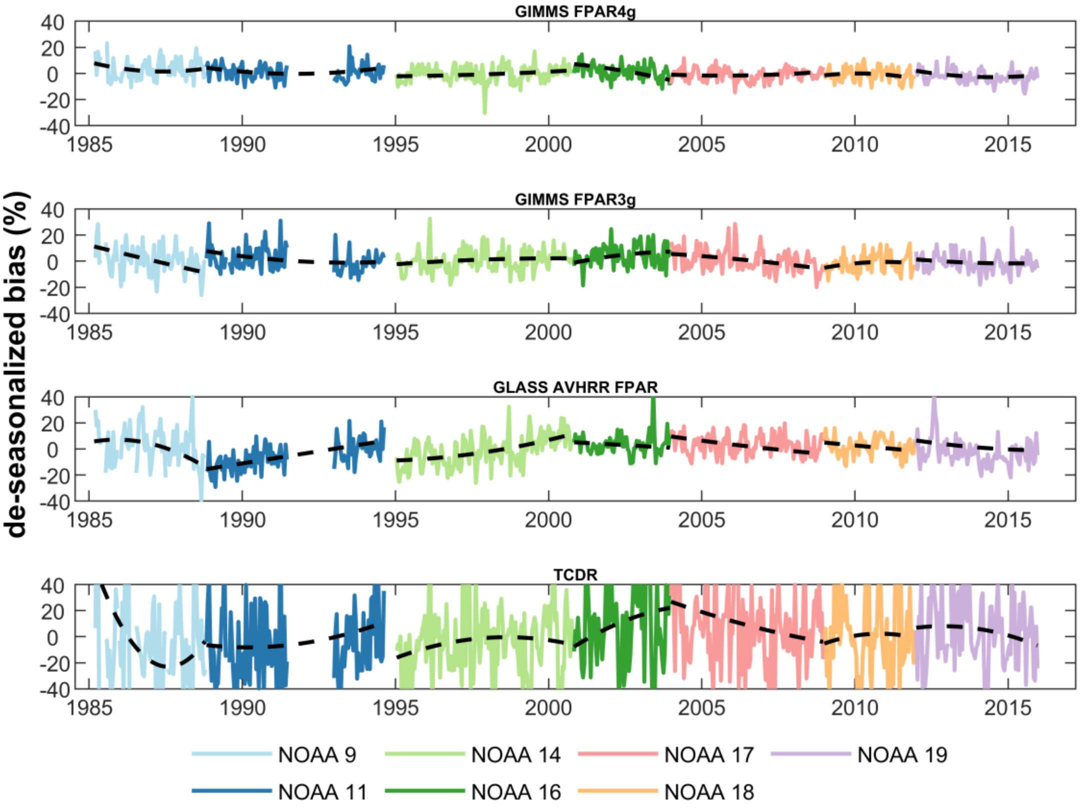

Fig. S11 Same as the Figure S9 but in CRO.

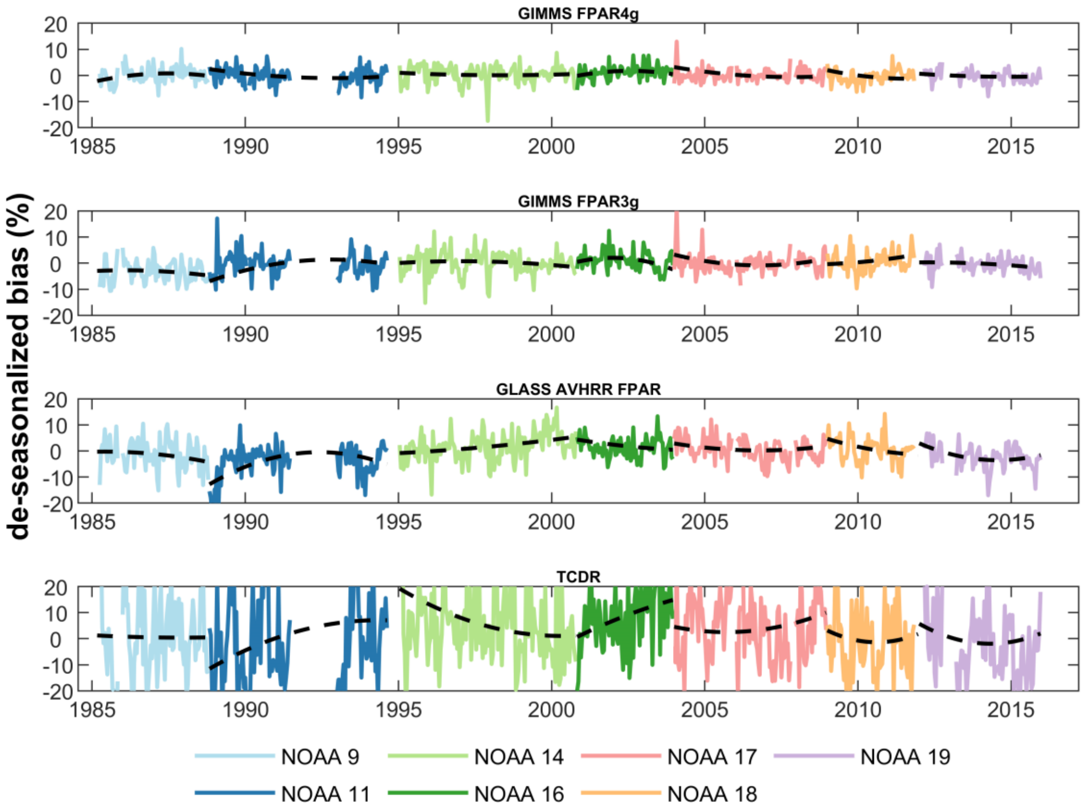

Fig. S12 Same as the Figure S9 but in SAV.

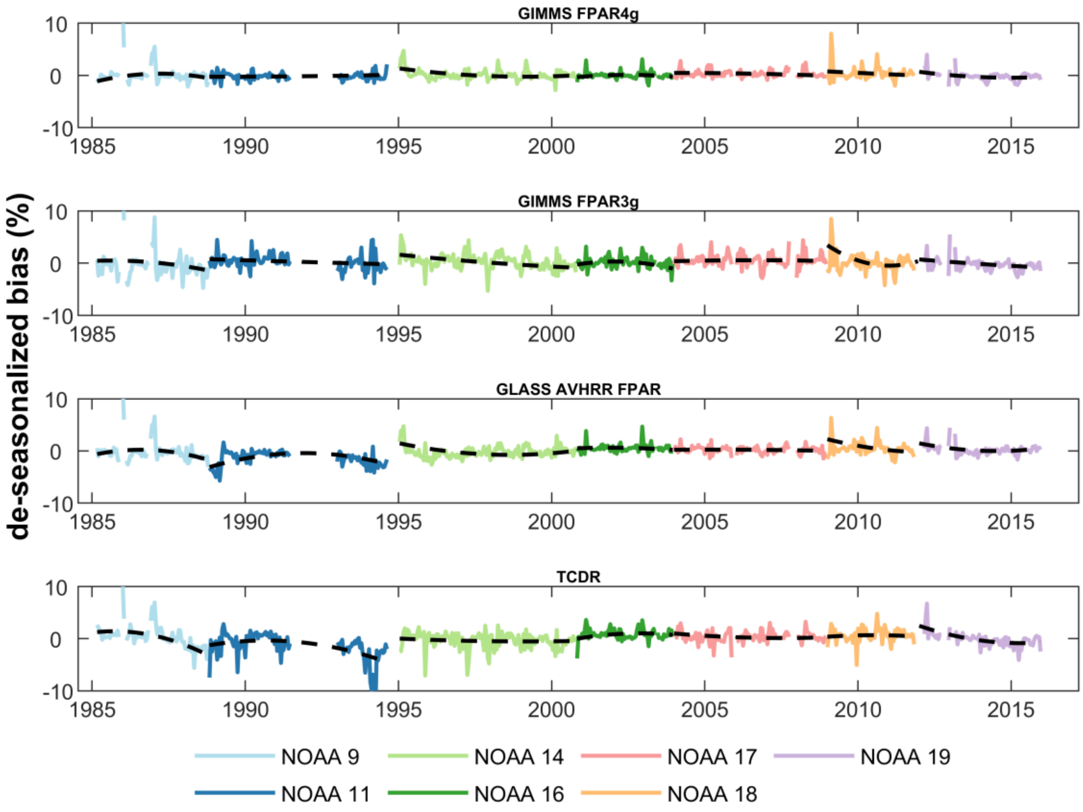

Fig. S13 Same as the Figure S9 but in EBF.

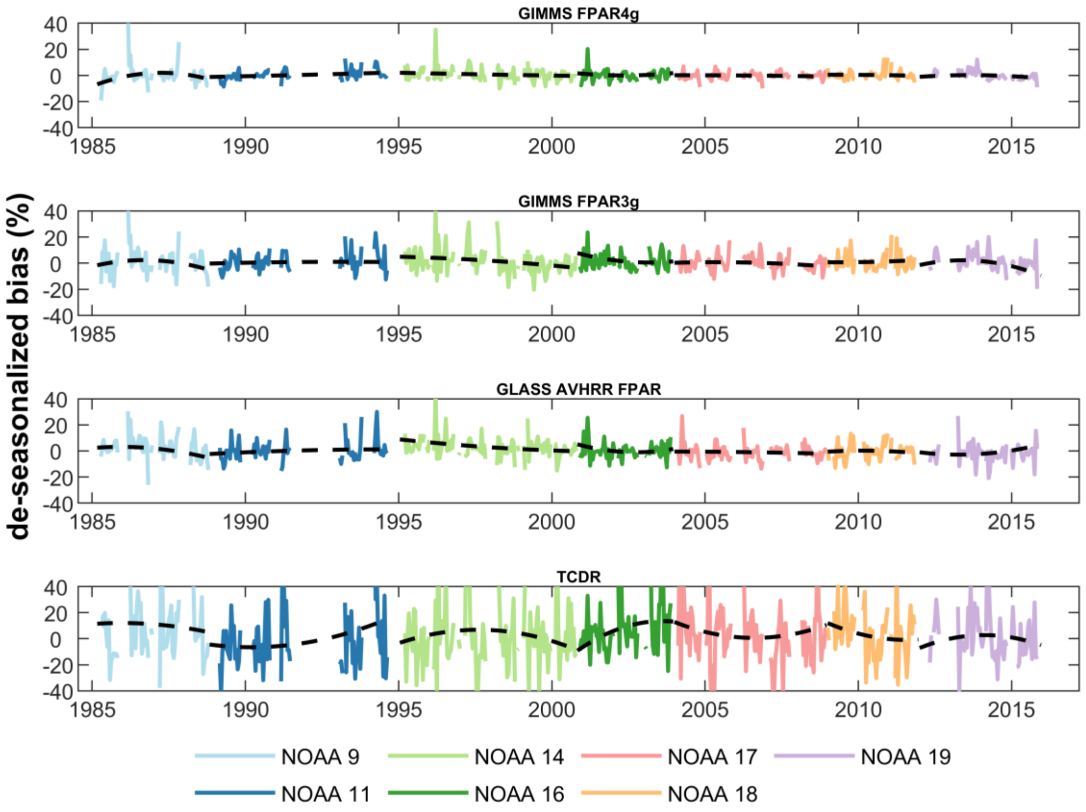

Fig. S14 Same as the Figure S9 but in DBF.

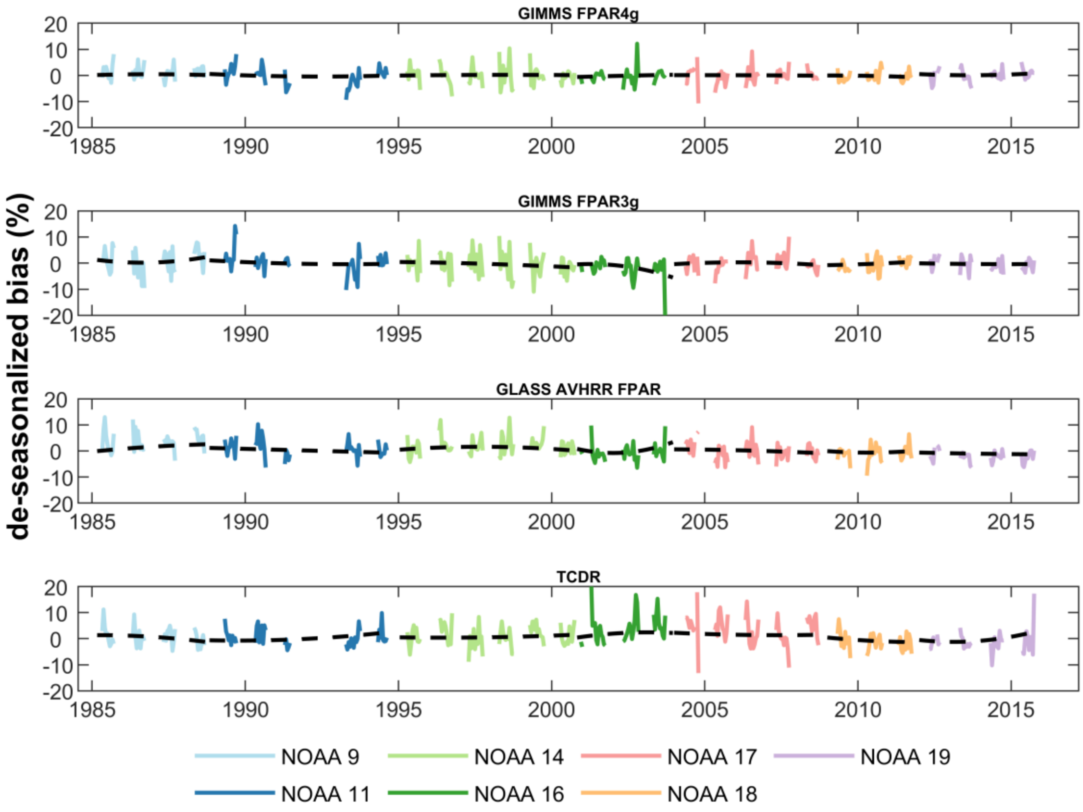

Fig. S15 Same as the Figure S9 but in ENF.

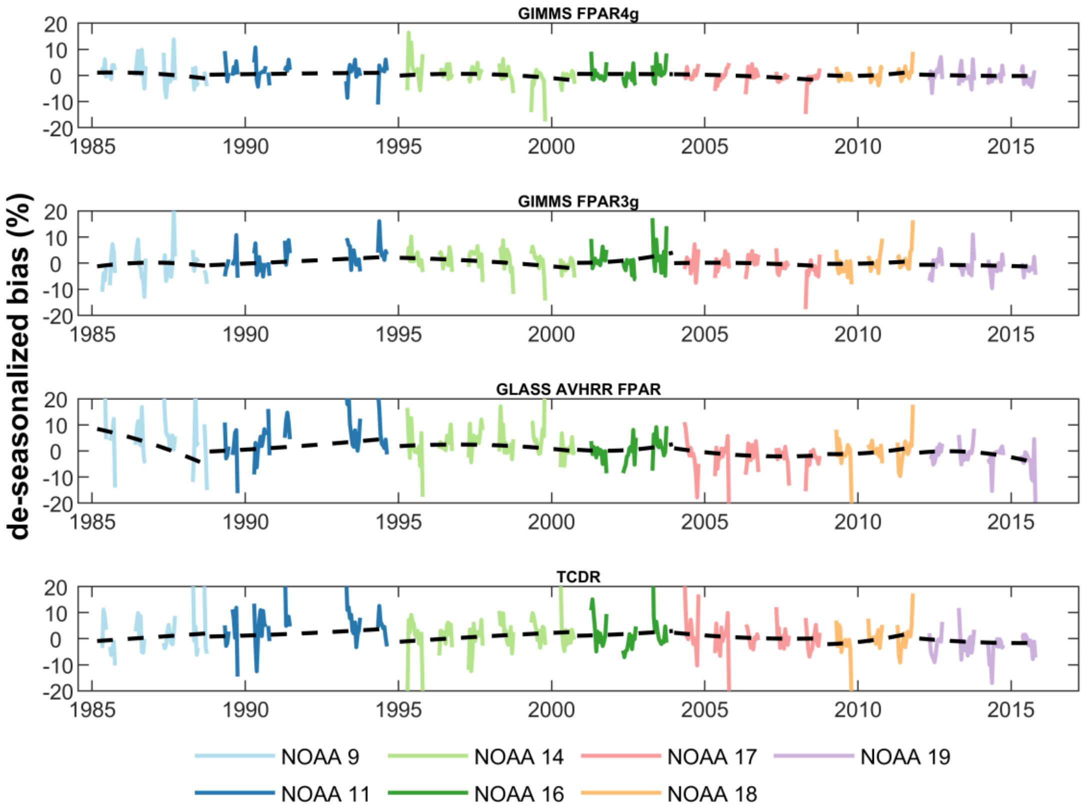

Fig. S16 Same as the Figure S9 but in DNF.

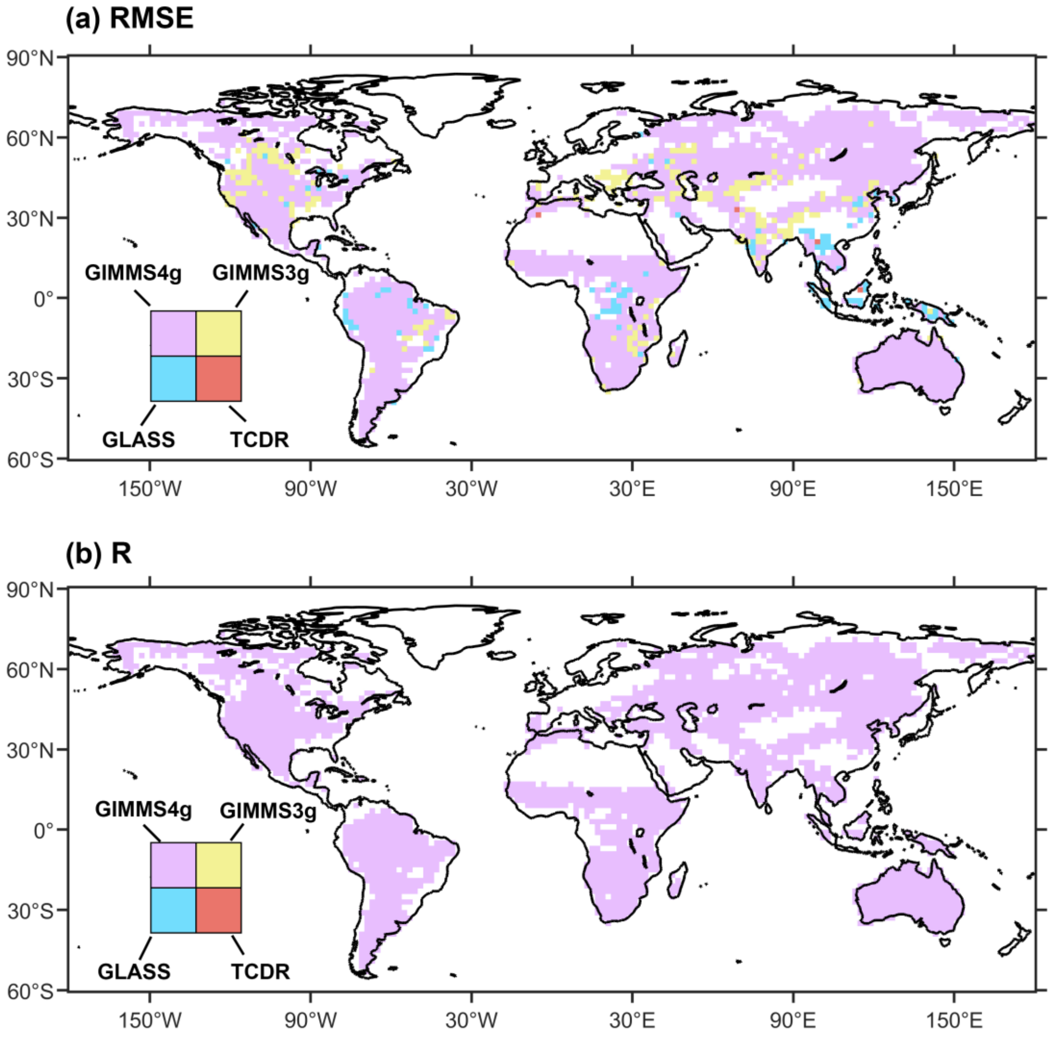

Fig. S17 Geographic distribution of FPAR products with (a) the smallest RMSE and (b) the largest R with the Landsat FPAR reference samples among four products.

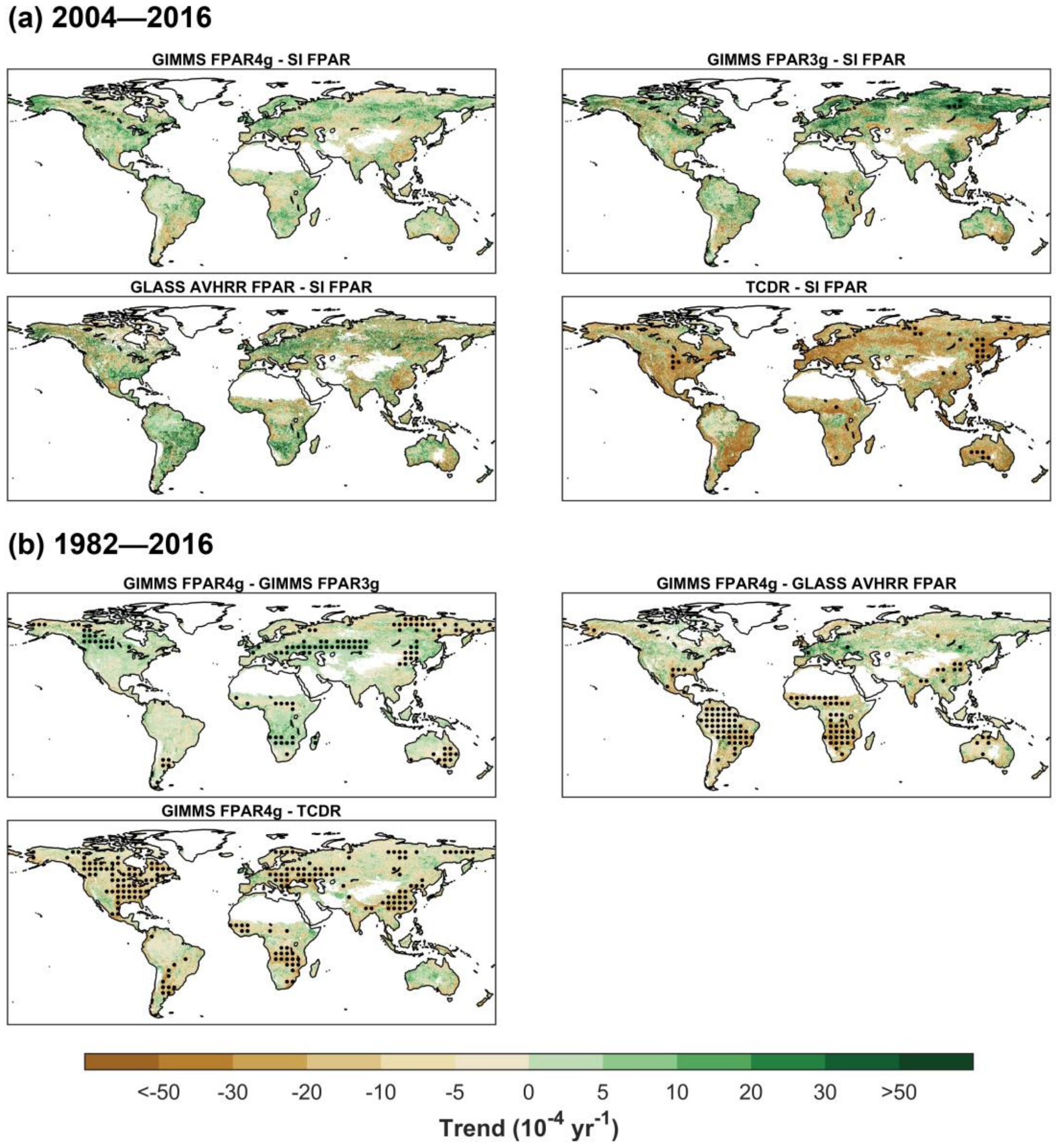

Fig. S18 Differences of the global FPAR annual linear trends between GIMMS FPAR4g and other FPAR products for (a) 2004—2016 and (b) 1982—2016. Regions marked with dots indicate their trends are statistically significant (Mann-Kendall test; $p<0.05$).

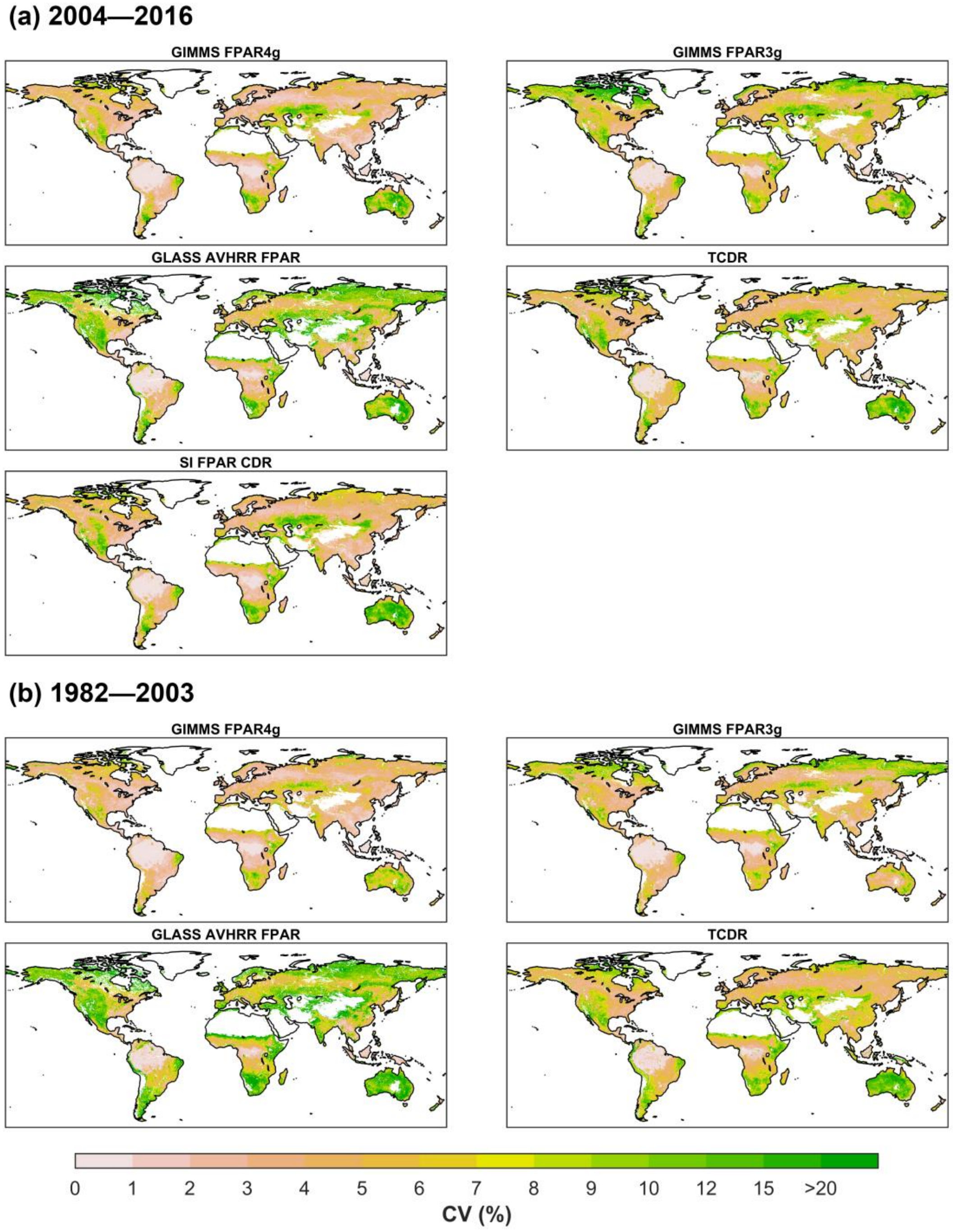

Fig. S19 Comparison of spatial patterns of interannual variability (IAV) of FPAR products. The coefficient of variation (CV) represents the IAV of FPAR. (a) 2004—2016. (b) 1982—2003.

Table S1. The sample size and optimal window size of the BPNN model for each half-month.

Half-month	EBF		Other Biomes	
	Sample Size	Window Size	Sample Size	Window Size
**1**	69828	11	39451	25
**2**	69927	11	42483	25
**3**	69876	11	46495	25
**4**	69558	11	47256	25
**5**	69855	11	47376	25
**6**	70023	11	47655	25
**7**	69978	11	48508	25
**8**	70134	11	51013	25
**9**	69819	11	54828	25
**10**	69777	11	57519	25
**11**	69654	11	60188	25
**12**	69666	11	61131	25
**13**	69924	11	61603	25
**14**	69840	11	61770	25
**15**	69591	11	61827	25
**16**	69921	11	62031	25
**17**	69798	11	61959	25
**18**	69837	11	61982	25
**19**	69882	11	58375	25
**20**	69948	11	53571	25
**21**	69963	11	47215	25
**22**	69876	11	41705	25
**23**	69990	11	37198	25
**24**	69852	11	34328	25

Table S2. Accuracy assessment metrics of the BPNN model for each half-month.

Half-month	Metrics			
	R^2	*RMSE*	*MAE*	*MAPE(%)*
**1**	0.98	0.04	0.03	4.29%
**2**	0.98	0.04	0.03	4.31%
**3**	0.98	0.04	0.03	4.39%
**4**	0.98	0.04	0.03	4.42%
**5**	0.98	0.04	0.03	4.41%
**6**	0.98	0.04	0.03	4.39%
**7**	0.98	0.04	0.03	4.33%
**8**	0.98	0.04	0.03	4.31%
**9**	0.98	0.04	0.03	4.34%
**10**	0.98	0.04	0.03	4.35%
**11**	0.98	0.04	0.03	4.36%
**12**	0.98	0.04	0.03	4.36%
**13**	0.97	0.04	0.03	4.36%
**14**	0.97	0.04	0.03	4.37%
**15**	0.97	0.04	0.03	4.38%
**16**	0.97	0.04	0.03	4.39%
**17**	0.97	0.04	0.03	4.40%
**18**	0.97	0.04	0.03	4.42%
**19**	0.97	0.04	0.03	4.41%
**20**	0.97	0.04	0.03	4.40%
**21**	0.97	0.04	0.03	4.39%
**22**	0.97	0.04	0.03	4.37%
**23**	0.97	0.04	0.03	4.35%
**24**	0.97	0.04	0.03	4.34%

Table S3. Accuracy assessment metrics for Random Forests models used to generate Landsat reference samples in eight biomes. R^2: R-squared; RMSE: root mean square error; MAE: mean absolute error; MAPE: mean absolute percentage error.

Metrics	Satellite/Sensor	Biome type							
		GRA	SHR	CRO	SAV	EBF	DBF	ENF	DNF
	**Landsat5/TM**	0.95	0.97	0.94	0.9	0.88	0.96	0.8	0.91
R^2	**Landsat7/ETM+**	0.95	0.98	0.93	0.91	0.89	0.96	0.79	0.9
	**Landsat8/OLI**	0.96	0.98	0.94	0.91	0.89	0.96	0.79	0.92
	**Landsat5/TM**	0.03	0.02	0.04	0.05	0.02	0.04	0.06	0.05
RMSE	**Landsat7/ETM+**	0.03	0.02	0.04	0.05	0.02	0.05	0.06	0.06
	**Landsat8/OLI**	0.03	0.02	0.03	0.05	0.02	0.04	0.06	0.05
	**Landsat5/TM**	0.02	0.01	0.03	0.04	0.01	0.03	0.04	0.04
MAE	**Landsat7/ETM+**	0.02	0.01	0.03	0.03	0.01	0.03	0.04	0.04
	**Landsat8/OLI**	0.02	0.01	0.02	0.03	0.01	0.03	0.04	0.03
	**Landsat5/TM**	8.51%	6.44%	9.45%	7.51%	0.90%	4.74%	5.50%	5.46%
MAPE	**Landsat7/ETM+**	8.28%	6.35%	9.09%	7.35%	1.04%	5.18%	5.76%	5.88%
	**Landsat8/OLI**	7.36%	5.37%	8.66%	6.85%	1.19%	3.74%	5.25%	4.63%
